# Biased sequential sampling underlies the effects of time pressure and delay in social decision making

**DOI:** 10.1038/s41467-018-05994-9

**Published:** 2018-09-03

**Authors:** Fadong Chen, Ian Krajbich

**Affiliations:** 1 School of Management, Zhejiang University, Hangzhou, 310058 China; 2 Neuromanagement Lab, Zhejiang University, Hangzhou, 310058 China; 3 Department of Economics, The Ohio State University, Columbus, OH 43210 USA; 4 Department of Psychology, The Ohio State University, Columbus, OH 43210 USA

## Abstract

Social decision making involves balancing conflicts between selfishness and pro-sociality. The cognitive processes underlying such decisions are not well understood, with some arguing for a single comparison process, while others argue for dual processes (one intuitive and one deliberative). Here, we propose a way to reconcile these two opposing frameworks. We argue that behavior attributed to intuition can instead be seen as a starting point bias of a sequential sampling model (SSM) process, analogous to a prior in a Bayesian framework. Using mini-dictator games in which subjects make binary decisions about how to allocate money between themselves and another participant, we find that pro-social subjects become more pro-social under time pressure and less pro-social under time delay, while selfish subjects do the opposite. Our findings help reconcile the conflicting results concerning the cognitive processes of social decision making and highlight the importance of modeling the dynamics of the choice process.

## Abstract (web summary)

It has been proposed that humans make unselfish decisions if constrained to decide quickly, but other research has suggested that time constraint makes us selfish. Here, the authors reconcile these two views showing that pro-social people become more pro-social under time pressure, but selfish subjects do the opposite.

## Introduction

Social decisions typically involve conflicts between selfishness and pro-sociality. A basic goal in decision science is to understand the cognitive processes that underlie these social decisions. There is a large literature describing the various factors that influence other-regarding behavior, including distributional preferences^1–3^, reciprocity^4^, social distance^5^, and guilt-aversion^6^, but these are all static models that simply predict choice outcomes. Recently there have been efforts to understand the dynamics of social decision making, with both single-process^7–9^ and dual-process^10–12^ models. However, the nature of social decision making is still disputed^7–24^.

One question is whether social decisions are the result of a single comparison process, or the result of two processes: one, a fast and intuitive process and the other, a slow and deliberative process^7,10^? The second question is whether people exhibit a selfish or pro-social bias? The latter question is usually posed under the presumption of dual processes: given that there is an intuitive process, does it favor selfishness or pro-sociality?

To answer this question, some dual-process researchers have examined relative response times (RT)^10,11,13,14,17–19,23^ to establish people’s intuitions. However, it has recently been argued that RT data cannot be used as evidence for intuitive/deliberative processes, since they are sensitive to the particular choice problems used by the researchers^7,16,25^.

An alternative approach which does not have this limitation is to experimentally manipulate RT (e.g., using time pressure) or impose cognitive load to try to establish people’s intuitive responses. In this manipulation literature, some have made a distinction between behavior in giving contexts (e.g., dictator games) and in cooperative contexts (e.g., public goods games).

In the giving context, subjects are simply asked whether they would like to give some of their money to another person (or charity). Here, some studies conclude that promoting intuition increases altruistic behavior^15^, while others find no effect of promoting intuition^20^. A meta-analysis finds that promoting intuition increases giving for women but not for men^26^.

In the cooperative context, subjects are put into groups of two or more and can pay a cost to give a larger benefit to the other(s) in their group. Here, some studies conclude that people’s intuition favors cooperation^10,24,27^, while other studies conclude that promoting intuition has no effect on cooperation^21,22,28^; a meta-analysis finds that intuition promotes cooperation^29^, and that—unlike for giving behavior—this is equally true for both women and men^30^. (One study in which participants were incentivized to make a choice quickly and then could subsequently change their decision did not find a difference between giving and cooperation^31^). Here we have decided to focus on the giving context, since these games are easier to interpret, as they do not depend on subjects’ beliefs about others’ choices.

Given the mixed evidence for whether intuition favors pro-sociality, cooperation, or selfishness, we return to the question of whether a single comparison process might better describe social decisions. A growing literature in decision neuroscience has argued for the prevalence of sequential sampling model (SSM) processes in decision making and cognition. These models, exemplified by the diffusion model (DDM), assume that information is sampled continuously until there is sufficient net evidence for one of the available options^32^.

One challenge to the simple SSM story in ref. ^7^ is the body of time pressure results reported in some articles^10,21,24,27,28^. If, indeed, time pressure amplifies existing behavioral tendencies^10^, an unbiased SSM (no starting point bias) cannot account for that behavior. In a SSM framework, time pressure reduces the amount of evidence needed to reach a decision, reducing RT but also consistency. In an unbiased SSM, reducing consistency should push the probability of any particular response towards 50%.

In some cases, decision makers may exhibit a bias towards one response, perhaps because that response consistently yields better outcomes. This behavior is captured by a bias in the starting point of the process^33–37^. In such cases, reducing the amount of evidence needed to reach a decision will amplify the choice bias. The starting point is therefore the natural candidate for explaining the purported effects of time pressure on social preferences.

We have two goals in this paper. The first is to document the effects of time-pressure and time-delay, using a wide array of decision problems and accounting for individuals’ social preferences. The second is to argue for a DDM with biased starting points (“biased DDM”), which integrates the dual process framework with the SSM framework and can account for both RTs and the effects of time manipulations. We provide a clear mechanism for how a predisposition (which some might call an “intuition”) and deliberation might interact to yield a decision. In sum, we aim to offer a unified account of social decision making that provides a clear explanation for the conflicting RT and time-pressure data in the literature.

We test our model using an experiment where subjects made binary decisions in a series of mini-dictator games, in time-free, time-pressure, and time-delay conditions. We take each subject’s change in pro-sociality from time-delay to time-pressure conditions as the measure of their predisposition. We find that people are heterogeneous in whether they are predisposed to pro-sociality (more pro-social under time pressure) or selfishness (more selfish under time pressure). In an out-of-sample test, this predisposition predicts subjects’ pro-sociality in the time-free condition. We then fit the biased DDM to the time-free data, show that it outperforms an unbiased DDM (and in some cases, a standard logistic choice model^8^), and find that subjects’ predispositions predict their starting points in the model. In particular, subjects with starting points biased towards pro-sociality become more pro-social under time pressure and more selfish under time delay, while subjects with starting points biased towards selfishness become more selfish under time pressure and more pro-social under time delay.

## Results

### The task

**Fig. 1:**
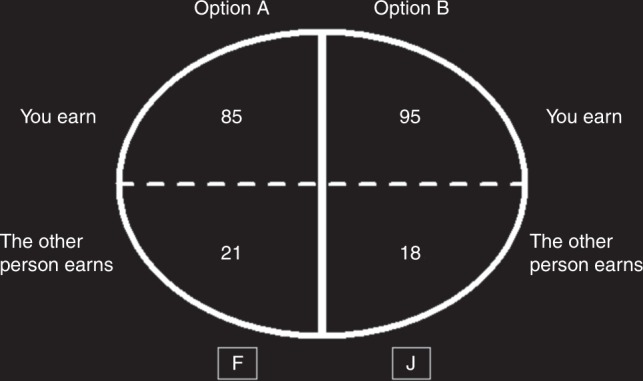
Screenshot of the mini-dictator game in the time-free condition. In this game, the subject can either press key “J” to choose the selfish option (Option B) which has a higher payoff for herself (95 vs. 85 in this example) or press key “F” to choose the pro-social option (Option A) which has a higher payoff for the other participant (21 vs. 18 in this example). The text is enlarged for display purposes. Refer to Supplementary Fig. 1 for actual screenshots

In the experiment, subjects made binary decisions in 200 mini-dictator games, where they allocated money between themselves (dictator) and another subject (receiver) anonymously. Each decision involved a conflict between selfishness and advantageous inequality aversion^2^ (Fig. 1, Supplementary Fig. 1). In other words, each game offered subjects the opportunity to increase the receiver’s earnings by reducing their own earnings, reducing the inequality between them (see Supplementary Note 1).

We divided the 200 trials into four blocks of 50 games each. In the time-pressure block, subjects had to make each decision within 2 s. In the time-delay block, subjects had to make each decision after viewing the options for 10 s. In the other two (time-free) blocks, subjects had unlimited time to make each decision. The first and last blocks were time-free blocks, while the other two were counterbalanced across subjects (see Methods, Supplementary Methods for more detail).

### The bias towards selfishness or pro-sociality

We employ the inequality aversion model proposed by Fehr and Schmidt^2^ to estimate subjects’ preferences (advantageous inequality aversion, *β*) under time-free (*β*_f_), time-pressure (*β*_p_), and time-delay (*β*_d_) conditions separately (see Methods). The estimation results show that the effect of decision time differs substantially across subjects. To see this, we split subjects according to the median indifference *β* (the *β* which would make a subject indifferent between the two options) from all of our choice problems; with this cutoff, 93% of the selfish subjects chose the selfish option on the majority of trials, while 100% of the pro-social subjects chose the pro-social option on the majority of trials (see Supplementary Note 2 for analyses based on other cutoffs).

**Fig. 2:**
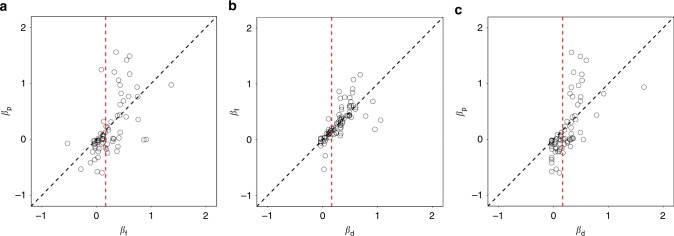
Advantageous inequality aversion (*β*) under different time conditions. Higher *β* indicates stronger pro-sociality. Each dot represents one subject’s degree of pro-sociality (*β*), comparing **a** time-free and time-pressure conditions, **b** time-delay and time-free conditions, and **c** time-delay and time-pressure conditions. Notice that subjects to the left of the vertical red line (split by the median indifference *β*, selfish subjects) are consistently shifted downwards while those to the right (pro-social subjects) are consistently shifted upwards. The number of subjects (*N*_S_) in the experiment was 102. 12 subjects whose *β*_f_ is outside of (−1, 2), 26 subjects whose *β*_p_ is outside of (−1, 2), and 8 subjects whose *β*_d_ is outside of (−1, 2) are not shown but were included in the statistical analyses. The black dashed line is the identity line

Subjects with higher *β*_f_ (pro-social subjects) became more pro-social under time pressure (*P* = 0.024, two-sided Wilcoxon signed-rank test, since *β* is not normally distributed), while subjects with lower *β*_f_ (selfish subjects) became more selfish under time pressure (*P* = 0.041) (Fig. 2a). Similarly, pro-social subjects became marginally less pro-social under time delay (*P* = 0.167), while selfish subjects became less selfish under time delay (*P* = 0.004), though these effects are less pronounced (Fig. 2b). The effect of decision time for prosocial and selfish subjects is more obvious if we compare time pressure and time delay conditions directly (Fig. 2c) (see Supplementary Table 1 for regression results).

In addition, *β*_p_−*β*_d_ is correlated with *β*_f_ (two-sided Spearman correlation test, *r* = 0.390, *P* = 5 × 10^−5^, Supplementary Fig. 2, Supplementary Tables 2-3). In other words, subjects were heterogeneous in the way in which reduced time affected their pro-sociality, and this effect predicted their pro-sociality under normal (time-free) conditions.

### Sequential sampling process

Prior studies have shown that behavior in social decision making is in line with SSM predictions^7–9^. Specifically, RT decreases with strength of preference. Our experiment not only allows us to test this hypothesis, but also allows us to test whether this hypothesis still holds under time pressure. In particular, if decisions under time pressure exclusively (or preferentially) rely on an intuitive, automatic process, then we might expect no (or a greatly reduced) relationship between RT and strength of preference.

**Fig. 3:**
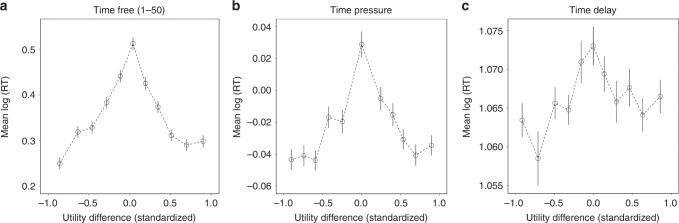
The relationship between log(RT) and utility difference (between Option A and Option B in the experiment) in the three time conditions. **a** The time-free condition, **b** the time-pressure condition, and **c** the time-delay condition. We standardize the utility differences to [−1,1] at the individual level (*N*_S_ = 102) and divide all the data in each time condition into 11 bins with equal size. Each circle represents one bin. Error bars represent standard errors across subjects. We only used 50 time-free trials (Games 1–50) to make the analysis comparable to the time-pressure and time-delay conditions, but we find similar results using all 100 time-free trials

To test this, we calculated the utility difference between Option A and Option B in the experiment (as an index of the strength of preference) using the estimated preference parameters (*β*_f_,*β*_p_,*β*_d_). Mixed-effects regressions with log(RT) as the dependent variable reveal that RT was negatively related with the absolute utility difference in in the time-free and time-pressure conditions (and marginally in the time-delay condition) (*t*(4997) = −9.71, *P* < 0.001 for the time-free condition, *t*(4997) = −7.186, *P* < 0.001 for the time-pressure condition, and *t*(4997) = −1.718, *P* = 0.086 for the time-delay condition) (Fig. 3). The relationship between RT and strength-of-preference is understandably weaker in the time-delay condition, since it is likely that in many cases subjects decided in under 10 s. In those cases, the true decision times are unobservable, and we should expect no relationship between RT and strength-of-preference.

### The biased DDM

**Fig. 4:**
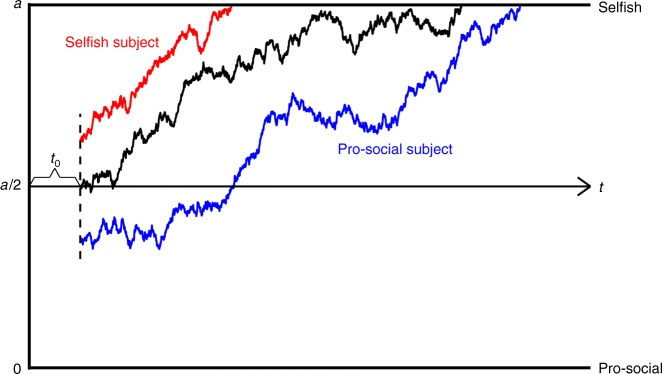
A graphical illustration of the DDM. 0 and *a* are the two preset thresholds for the pro-social option and the selfish option, and *a* is a free parameter. Non-decision time, *t*_0_, denotes the time required for encoding the stimulus and decision execution. The three paths indicate the evolution of the relative evidence (*R*) over time. The red path which starts near the threshold of the selfish option represents the DDM process for a subject with a response bias towards the selfish option; the blue path which starts near the threshold of the pro-social option represents the DDM process for a subject with a response bias towards the pro-social option; and the black path represents a DDM process without starting point bias (unbiased DDM)

These results paint a complex picture. On the one hand, the relationship that we observe between RT and strength of preference in all time conditions is consistent with a single SSM process. On the other hand, the amplification of preferences under time pressure (and attenuation under time delay) is the opposite of what one would expect from time pressure in an unbiased SSM. Here, we argue that a SSM with starting points biased towards subjects’ generally preferred actions can account for these patterns (Fig. 4).

To make things more concrete, we focus on the DDM^32^, which was originally developed for memory, cognition, and perception and has been increasingly used to study economic decision making^7,8,38–45^. The DDM assumes that decisions are generated by a noisy process that accumulates relative evidence (*R*) that one option is better than the other. The relative evidence *R* follows a diffusion process and evolves in small time increments according to a stochastic difference equation, *R*_*t*+1_ = *R*_*t*_ + *v* + *s*_*t*_ (with discrete time this is technically a random walk model), where *v* is the drift rate that represents the average strength of preference for the selfish option, and *s* represents mean-zero Gaussian noise. A choice is made once *R *reaches one of the two thresholds, normalizing the pro-social threshold to zero and the selfish threshold to a constant, *a*. An additional feature of the DDM is that there can be an initial bias in the starting point (*R*_0_), often referred to as a response bias^46^, towards selfishness or pro-sociality.

For now, we will assume that these starting points are biased towards a subject’s generally preferred choice; later we will verify this assumption by fitting the starting points to the data. Specifically, the process starts near the selfish threshold for subjects who are generally selfish (*R*_0_ > *a*/2), and the process starts near the pro-social threshold for subjects who are generally pro-social (*R*_0_ < *a*/2).

To see why starting point biases are necessary to account for our choice data, let’s first consider a simple DDM without starting point biases (unbiased DDM). In that model, drift rate is the sole determinant of “preference” in a given choice situation, where we define preference as the option that the subject would choose given unlimited time to decide. In our setting, the drift rate determines whether the subject is more than 50% likely to choose the selfish option, with positive drift rates producing predominantly selfish choices and negative drift rates producing predominantly pro-social choices. For a given drift rate, the threshold separation determines the subject’s preference-choice consistency. With infinite threshold separation, the subject would always choose in line with their preference, while with zero threshold separation, the subject would choose randomly.

In the DDM literature, time pressure is modeled using narrower decision thresholds (or in the case of time limits, collapsing thresholds), which reduce RT at the cost of consistency. This assumption is supported by a large body of research showing that time pressure narrows thresholds but does not affect drift rates^32,47,48^. Whether thresholds also collapse over time is an active debate in the DDM literature, with conflicting theoretical and empirical arguments^49–53^.

**Fig. 5:**
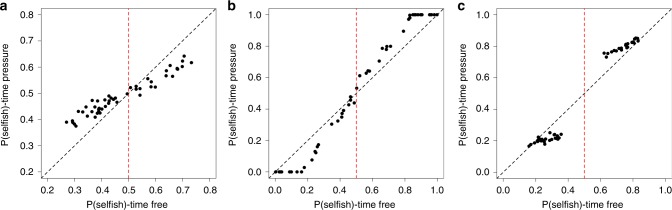
Simulations of the (biased) DDM with and without time pressure. **a** The simple DDM with no starting point biases, **b** the DDM with starting point biases proportional to drift rate, and **c** the DDM with a fixed starting point bias of +/− 0.25. Each dot represents one simulated subject. Note that in **a** the dots fall between the diagonal (black dashed line) and the horizontal midline, indicating that under time pressure these subjects’ choices move towards chance, while in **b** and **c** the dots fall between the diagonal and the vertical midline (red dashed line), indicating that under time pressure these subjects’ choices become more extreme. Only this latter pattern is consistent with the results seen in Fig. 2

To visualize the effects of time pressure on choice behavior in the unbiased DDM, we simulated 50 fake subjects with drift rates sampled from a uniform distribution *v*_*i*_∈[−0.0002, 0.0002]ms^−1^, and with a threshold separation of either *a* = 2 or *a* = 1. For each fake subject, we simulated 1000 trials with each threshold separation. What the simulations clearly show is that these fake subjects’ selfish choice probabilities are consistently closer to 50% with the narrower thresholds, i.e. under time pressure (Fig. 5a; also with collapsing thresholds, see Supplementary Fig. 3a). This is opposite to the pattern we see in our data.

Now let’s consider what happens when subjects’ decisions are partly determined by starting points. With infinite threshold separation, the starting point would have no effect and the subject would always choose in line with their preference. However, with finite threshold separation, the narrower the thresholds, the more influence the starting points have on the decision.

To visualize the effects of time pressure on choice behavior in the DDM with biased starting points (biased DDM), we again simulated 50 fake subjects with the same distribution of drift rates and threshold separations as before. We additionally assumed that each fake subject’s starting point (relative to *a*/2) was proportional to their drift rate. Specifically, a fake subject’s starting point was *R*_0_=*a*/2 + 5000·*v*. As before, for each fake subject, we simulated 1000 trials with each threshold separation. What these simulations clearly show is that the fake subjects’ choice probabilities are consistently more extreme with the narrower thresholds, i.e., under time pressure (Fig. 5b; also with collapsing thresholds, see Supplementary Fig. 3b). This is the pattern we see in our data.

These latter simulations assume that a subject’s starting point is proportional to their drift rate. We believe this to be a reasonable assumption, since subjects with more extreme preferences will find themselves more often making the same choice (either selfish or pro-social) and so may want to adjust their starting points further in that direction, to save time. Indeed, this is a likely mechanism for how people generate predispositions. Below, we verify this assumption by showing that *β*_f_ correlates with starting points. Nevertheless, a simpler model, for example *R*_0_ = *a*/2 ± 0.25, produces the same phenomenon of more extreme choice probabilities with narrower thresholds, but displays a discontinuity in choice behavior, due to the starting-point discontinuity at *v* = 0 (Fig. 5c; also with collapsing thresholds, see Supplementary Fig. 3c).

The simulations above are simplified in the sense that they assume a single drift rate (i.e., strength-of-preference) per subject. In the real experiment, each subject experienced a variety of decisions and therefore a variety of drift rates. Therefore, as a robustness check, we carried out the simulations behind Fig. 5 using the actual parameters estimated from the time-free data. We then compared the resulting *β*_f_ and *β*_p_ estimated from the simulated data. The biased DDM simulations (Supplementary Fig. 4a and Supplementary Fig. 4c) produced similar patterns as seen in the data (Figs. 2,  5). That is, the results show that under time pressure, simulated selfish subjects (split according to median indifference *β*) became more selfish and simulated pro-social subjects became more pro-social. Similar to Fig. 5a, the pattern produced by the unbiased DDM simulations (Supplementary Fig. 4b and Supplementary Fig. 4d) is not consistent with the results seen in the experiment.

In sum, if the only difference between selfish and pro-social subjects was their drift rates, then time pressure should have brought their behavior closer together (not observed in the data), but if they also differed in their starting points, then time pressure should have made their behavior more extreme (observed in the data). Differences between groups in other parameters might additionally be present, but they cannot explain the time-pressure phenomenon without biased starting points, since the effects of narrower (or collapsing) thresholds on choice (Fig. 5a, Supplementary Figs 3a, 4b, d) do not depend on the initial threshold separation or drift rates.

In the next section, we attempt to verify these conclusions with formal model fits on data independent from the data used to classify subjects as being predisposed to selfish or pro-social behavior. Specifically, we separate subjects based on whether they became more or less pro-social under tighter time constraints. We hypothesized that both groups of subjects would be better fit by DDMs with biased starting points than ones without. We additionally hypothesized that the subjects who became more pro-social under tighter time constraints would exhibit starting points biased towards the pro-social threshold, while subjects who became more selfish under tighter time constraints would exhibit starting points biased towards the selfish threshold.

### Model fitting

We first split subjects based on how their preferences changed from time-pressure to time-delay conditions, resulting in 56 selfishly predisposed subjects (*β*_p_ < *β*_d_) and 46 pro-socially predisposed subjects (*β*_p_ > *β*_d_). Splitting by gender, 24 of 56 (43%) females and 22 of 46 (48%) males were pro-socially predisposed, in contrast to the findings from ref. ^26^

We fit the DDM at both the group and individual level on the time-free data. We focus exclusively on fitting the time-free data since these are the only data that display the roughly log-normal RT distributions produced by the DDM. The time constraints in the other two conditions distort the RT distributions and preclude fitting DDMs to those data (Supplementary Fig. 5).

Our hypothesis was that the relative starting point, *z* = *R*_0_/*a* (*z*∈[0,1]), would be greater (less) than 0.5 for the selfishly predisposed (pro-socially predisposed) subjects. The starting point *z* was indeed greater than 0.5 (0.547 at the group level, and an average of 0.564 at the individual level) for selfishly predisposed subjects, and was less than 0.5 (0.403 at the group level and an average of 0.452 at the individual level) for pro-socially predisposed subjects (Table 1, Supplementary Table 4). At the individual level, the starting points were greater than 0.5 for 40 of 56 selfishly predisposed subjects (*P* = 0.002, two-sided Binomial test), and the starting points were less than 0.5 for 30 of 46 pro-socially predisposed subjects (*P* = 0.054) (see Supplementary Table 4 and Supplementary Note 3).

**Table 1:** Estimation results of the biased DDM

	Subject type	Relative starting point (*z*)	Non-decision time (*t*_0_) (s)	Threshold (*a*)
Group level	Selfishly predisposed	0.547 (0.010)	0.272 (0.076)	3.419 (0.047)
	Pro-socially predisposed	0.403 (0.008)	0.426 (0.042)	3.818 (0.061)
Individual level	Selfishly predisposed	0.564 (0.015)	0.743 (0.056)	3.356 (0.107)
	Pro-socially predisposed	0.452 (0.019)	0.692 (0.042)	4.204 (0.216)

The standard errors of the estimators are reported in parentheses. The standard errors at the group level are calculated using a jackknife method^74, 75^. The estimated drift rates and variability of the starting points at the group level are not reported. Details of the estimated parameters at the individual level are reported in Supplementary Table 4.

**Fig. 6:**
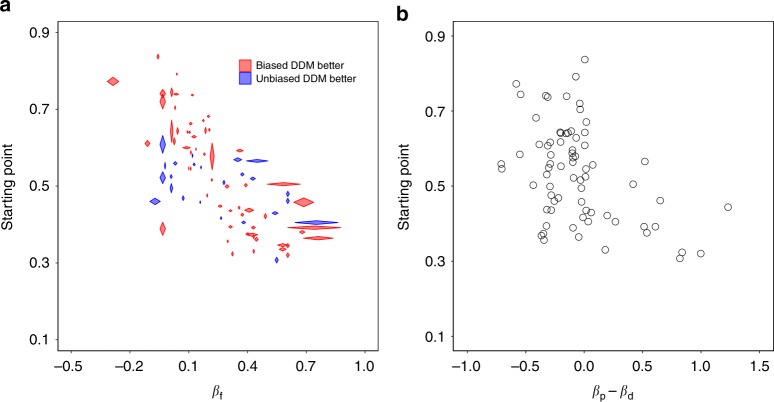
Starting point plotted against *β*_f_ and *β*_p_−*β*_d_. **a** Starting point vs. *β*_f_, and **b** starting point vs. *β*_p_−*β*_d_. Each diamond in **a** and each circle in **b** represents one subject. The length of the horizontal diagonal of each diamond in **a** represents the standard error (SE) of the *β*_f_, and length of the vertical diagonal represents the standard error of the starting point. Red represents subjects who were better fit with the biased DDM, while blue represents subjects who were better fit with the unbiased DDM. For illustration purposes, we constrain −1 < *β*_f_ < 1 and SE(*β*_f_) < 0.3 for **a** (82 of 102 subjects), and we constrain *β*_p_ and *β*_d_ to [−1, 2] for **b** (73 of 102 subjects), but we included all the 102 subjects in the statistical analysis

In the previous section, we assumed that starting points would be correlated with subjects’ generally favored options. Verifying this assumption, we found that the starting points were negatively correlated with *β*_f_ (two-sided Spearman correlation test, *r* = −0.594, *P* = 10^−11^, Fig. 6a). Importantly, starting points were also negatively correlated with *β*_p_−*β*_d_ (*r* = −0.460, *P* = 10^−6^, two-sided Spearman correlation test) (Fig. 6b, Supplementary Fig. 6). In other words, subjects who were more pro-social under time pressure compared to time delay, showed a larger starting point bias towards the pro-social threshold in the time-free condition. This indicates that we can use starting points, estimated on time-free data, to expose underlying biases which in the past have been inferred by comparing time-constrained conditions.

Finally, a logistic regression of *β*_p_−*β*_d_ on DDM parameters (Supplementary Table 5) revealed that the only significant predictor was the starting point bias (*P* = 0.006). This indicates that the starting point bias is likely the key mechanism to explain the impact of time constraints.

### Model validation

When fitting models to data, there is always a concern of over-fitting. That is why we have focused on the relationship between time-free model parameters and behavior in the time-pressure and time-delay conditions. Taking this idea a step further, in this section we test whether the time-free model with biased starting points (biased DDM) provides better fits after accounting for the number of model parameters (using BIC) and whether it can better predict other out-of-sample time-free data.

Looking at the individual-level fits, the BICs of the biased DDM were lower than the BICs of the unbiased DDM for 71 (of 102) subjects (two-sided Binomial test, *P* < 10^−4^). That is, the biased DDM generally fits the data better than the unbiased DDM. Specifically, the biased DDM fits the data better than the unbiased DDM for subjects who have a larger starting point bias, while the unbiased DDM fits the data better than the biased DDM for subjects whose starting point is near 0.5 (Fig. 6a).

Next we validate the biased DDM by comparing its out-of-sample predictions with those of the unbiased DDM and logistic choice models^54,55^. In one logistic model (Logit), the dependent variable was a dummy indicating whether the choice was selfish or pro-social, and the independent variables were the difference between the dictator’s payoffs (DicDiff) and the difference between the receiver’s payoffs (ReceDiff). In a second logistic model (Logit+RT), we added another independent variable, RT. More specifically, we estimated these models for selfishly predisposed and pro-socially predisposed subjects separately using one half of the data (Games 1–50) and used the estimated parameters to predict choices in the other half of the data (Games 51–100, see Methods). We then calculated the absolute error (AE) between the predicted and empirical probabilities of choosing the selfish option in each game (Table 2; see also Supplementary Figs 7–9, Supplementary Table 6).

**Table 2:** Out-of-sample prediction results

	Selfishly predisposed subjects	Pro-socially predisposed subjects
∑AE_biased DDM_	3.147	3.445
∑AE_unbiased DDM_	3.575	4.091
∑AE_Logit_	3.081	3.757
∑AE_Logit + RT_	3.127	3.749
Cramer’s *λ* of biased DDM	0.198	
Cramer’s *λ* of unbiased DDM	0.163	
Cramer’s *λ* of Logit	0.1648	
Cramer’s *λ* of Logit+RT	0.1650	

In this table, we use the data from Games 1–50 to predict decisions in Games 51–100 in the time-free condition

The summed AE for the biased DDM was less than that for the unbiased DDM for both selfishly predisposed and pro-socially predisposed subjects. Since the number of selfishly predisposed and pro-socially predisposed subjects was not equal, we also used Cramer’s *λ*^56^ to quantify each model’s predictive power (higher *λ* = better predictions). Cramer’s *λ* for the biased DDM (0.198) was higher than that of the unbiased DDM (0.163), Logit (0.1648), and Logit+RT (0.1650). Therefore, the biased DDM generally outperformed the other models in terms of out-of-sample predictions.

## Discussion

Our paper provides an alternative account for the cognitive processes underlying social decision making. Subjects are heterogeneous in whether they generally favor selfishness or pro-sociality. This produces a bias in their initial belief that the selfish or pro-social option is the better choice. Once the options appear, subjects update their initial beliefs by evaluating and comparing the options, in line with a SSM account. Thus, a DDM with biased starting points unifies single- and dual-process accounts of social decision making, allowing us to explain features of the data, and other findings in the literature, that otherwise could not be explained by either account on its own. In particular, it captures the relationship between strength-of-preference and RT, while also explaining why choice biases are magnified under time pressure and attenuated under time delay. Other evidence-accumulation models that are designed to capture intuition^57,58^ do not explain these results. The model in ref. ^57^ does not explicitly predict any effects of time pressure or time delay, and the model in ref. ^58^ predicts a bi-modal RT distribution, which is not the case in our data (Supplementary Fig. 5).

Our out-of-sample prediction results reveal that the biased DDM outperforms the simple unbiased DDM and logistic choice models in predicting choices. Thus, it is important to take these prior biases into account when modeling social decision making. These results also underline the usefulness of computational models for describing social behavior^59,60^. In particular, what we are suggesting is that time pressure affects decisions not by engaging a different decision process, but instead by simply allowing less time for the decision maker to update from their prior. Thus, under time pressure, the prior plays a larger role in determining the decision.

One might wonder how a starting point would be biased towards the selfish or pro-social option? Starting-point biases are more often associated with response biases (e.g., spatial biases) and it is not immediately obvious how this would translate to a setting where the alternatives vary across trials. One likely possibility is that subjects initially scan their own payoffs to determine which option is better for them, consistent with ref. ^17^. This is consistent with the idea that most of the non-decision time is for stimulus encoding.

An advantage of our approach is that it relies on a well-established modeling framework that has proven useful in many domains of human behavior^61,62^ and that has substantial support from neural data^63–66^. Moreover, the consequences of starting point biases in SSMs are mathematically precise and well understood, generating falsifiable predictions that can be tested with choice and RT distributions. Although here we have restricted ourselves to the dictator game, this framework could be applied to cooperative settings, provided that beliefs could be measured or estimated, and utilities calculated. This would be a useful next step for this research.

We acknowledge that the direct evidence supporting a DDM-like process under time delay is relatively weak. While we believe that a DDM process is still at work under time delay, the issue is that the true RTs are unobservable. That is, we do not know when subjects actually make their decisions, since they are forced to wait until 10 s have passed before responding. It seems likely that most subjects still do use a DDM, raise their decision thresholds to allow for the extra time, but still finish their decisions well before they are cued to respond. After the cue, they respond at a roughly random time. This is consistent with the roughly Normal RT distribution seen in this condition (Supplementary Fig. 5). It is worth nothing though that the biased DDM simulations under time delay do produce similar choice patterns (Supplementary Fig. 10) as seen in the experimental data (Fig. 2b).

The SSM framework also opens the door for more detailed modeling of dual process cognition, building on other implementations of intuition vs. deliberation^57,67–70^. Finally, a starting point bias captures the behavioral phenomenon while being agnostic about its source (e.g., genetics, upbringing, experiment instructions, prior decisions, etc.). More research is required to fully characterize the factors that affect these starting points and how they change over time^71^.

## Methods

### Subjects

In total 102 subjects (56 females) participated in the experiment. Eighteen subjects took part in an initial experiment at The Ohio State University (OSU), followed by 84 subjects at the University of Konstanz. On average, subjects earned 20 dollars at OSU and 16 Euros at Konstanz (including show-up fees). Subjects gave informed written consent before receiving the instructions at OSU, and we obtained informed consent from subjects when they registered for the experiment at Konstanz. OSU’s Human Subjects Internal Review Board approved the experiment.

### Experimental design

The mini-dictator games under different time conditions had the same properties but minor differences in payoffs. Specifically, the differences between the dictators’ payoffs (DicDiff) were 2, 4, 6, 8, and 10, while the differences between the receivers’ payoffs (ReceDiff) were from 3 to 57, in steps of 6. In every trial, the subject had to decide whether to give up some of their own money in order to increase the other subject’s payoff and reduce the inequality between them. We first fixed the parameters for 50 games in the time-free condition (Games 1–50). We then decreased (increased) all the payoffs by 1 for one half of these games and increased (decreased) all the parameters by 1 for the other half of games to get the 50 games for the time-pressure (-delay) conditions. Finally, we decreased all the parameters by 2 for one half of the games and increased all the parameters by 2 for the other half of games to get the other 50 time-free trials (Games 51–100).

At the beginning of each session, we randomly matched subjects into two-person groups. We randomized the order of the games within the different time conditions for each group.

### Preference estimation

We employ the inequality aversion model proposed by Fehr and Schmidt^2^ to estimate subjects’ preferences using maximum likelihood estimation (MLE). A subject’s utility for each option in the mini-dictator game is given by

1 $$U\left( {u_{\mathrm{d}},u_{\mathrm{r}}} \right){\mathrm{ = }}u_{\mathrm{d}} - \beta \left( {u_{\mathrm{d}} - u_{\mathrm{r}}} \right),$$

where *u*_d_ is the dictator’s payoff and *u*_r_ is the receiver’s payoff. The parameter *β* indicates the subject’s social preference, with higher *β* indicating stronger pro-sociality.

### Fitting the biased DDM at the group level

We estimated the biased DDM using subjects’ 100 decisions in the time-free condition for selfishly predisposed and pro-socially predisposed subjects separately. We used Fast DM^72^ with the Kolmogorov-Smirnov method to estimate the model. In the estimation, we let the drift rate (*v*) depend on the payoffs in each trial. Thus, we estimated a drift rate for each combination of DicDiff and ReceDiff. Since we had 50 different combinations (5 DicDiff and 10 ReceDiff) in our games, this meant 50 drift rates. In the estimation, we also included inter-trial variability of the starting point (szr), but kept szr, the non-decision time (*t*_0_), and the threshold (*a*) constant across games.

### Fitting the DDM at the individual level

We estimated the biased DDM and the unbiased DDM at the individual level using subjects’ 100 decisions in the time-free condition. We used RWiener^73^ with MLE to estimate the model. In the estimation, we set the drift rate (*v*) as a linear function of DicDiff and ReceDiff,

2 $$v = d_{\mathrm{c}} + d_{\mathrm{d}} \ast {\mathrm{DicDiff}} + d_{\mathrm{r}} \ast {\mathrm{ReceDiff}}$$

Thus, we estimated six parameters in total for the biased DDM: the relative starting point (*z*), the threshold (*a*), the non-decision time (*t*_0_), the drift constant (*d*_c_), the weight on DicDiff (*d*_d_), and weight on ReceDiff (*d*_r_). In the unbiased DDM, we fixed the relative starting point at *z* = 0.5. Supplementary Table 4 and Supplementary Table 7 show the details of the estimation results. The BIC for each estimation is given by

3 $${\mathrm{BIC = }}\ln \left( n \right)k - 2{\mathrm{ln}}(L),$$

where *n* is the number of observations in the data, *k* is the number of parameters estimated by the model, and *L* is the likelihood in the MLE estimation.

### Nonparametric test

We use Spearman correlation tests whenever looking at the correlation between *β* and other measures/parameters. The fitting procedure for Fehr-Schmidt model can produce extreme parameter values for some subjects who (almost) always choose the pro-social or selfish options. For example, some subjects have *β* of 335 or −217, while the typical range of values is between 0 and 1. For a linear Pearson correlation, this can seriously distort the estimates. The rank-based Spearman correlations allow us to include all subjects, and the resulting correlation is almost the same as the Pearson correlation where we exclude these “outlier” subjects.

### Out-of-sample predictions

To do out-of-sample predictions, we estimated the biased DDM and the unbiased DDM based on half of the data in the time-free condition, and then used the estimated parameters to predict subjects’ decisions in the other half of the data. Since we did not have enough trials at the individual level, we estimate these models at the group level. Specifically, we estimated the model using Games 1–50 and 51–100 separately. Here we again used the Kolmogorov-Smirnov method of Fast DM^72^ (the estimation results are shown in Supplementary Table 8). We used the estimated parameters to simulate the biased DDM and the unbiased DDM 5000 times for each game to determine the predicted probability of the selfish choice in each game.

For the logistic model predictions, we regressed the following two logistic models on the same halves of the data (the regression results are shown in Supplementary Table 9),

4 $${\mathrm{Logit:Selfish = }}\gamma _0 + \gamma _1 \ast {\mathrm{DicDiff}} + \gamma _2 \ast {\mathrm{ReceDiff}} + \varepsilon ,$$

5 $$\begin{array}{l}{\mathrm{Logit + RT:Selfish}}\\ {\mathrm{ = }}\gamma _0{\mathrm{ + }}\gamma _1 \ast {\mathrm{DicDiff}}{\mathrm{ + }}\gamma _2 \ast {\mathrm{ReceDiff}}{\mathrm{ + }}\gamma _3 \ast {\rm RT}{\mathrm{ + }}\varepsilon ,\end{array}$$

and used the results to calculate the predicted probabilities of selfish choices.

Since the number of selfishly predisposed subjects was different from the number of pro-socially predisposed subjects, we measured the aggregate predictive performance using Cramer’s *λ*^56^ which is calculated as

6 $$\lambda {\mathrm{ = }}\bar P^ + - \bar P^ - ,$$

where $\bar P^ +$ and $\bar P^ -$ denote the predicted probability of choosing the selfish option on trials in which the selfish option was actually chosen and on trials in which the pro-social option was actually chosen, respectively. Thus, *λ*∈[0,1] reflects how much of the choice variation across trials is captured by the model. *λ* = 1 indicates that the model can perfectly predict choice outcomes, while *λ* = 0 indicates that the model predicts decisions at chance.

### Code availability

The code for the analyses presented in this article is available from the corresponding author upon reasonable request.

## Electronic supplementary material

Supplementary Information

## Data Availability

The data that support the findings in this article are available in OSF with the identifier 10.17605/OSF.IO/UKG7B.
