# Supplementary material for: Biased sequential sampling underlies the effects of time pressure and delay in social decision making
Source: Nat Commun. 2018 Sep 3;9:3557. doi: 10.1038/s41467-018-05994-9 (PMC6120923; doi:10.1038/s41467-018-05994-9)
Supplement: Supplementary file 1 — Supplementary Information [file 41467_2018_5994_MOESM1_ESM.pdf]

Supplementary Information

Biased sequential sampling underlies the effects of time pressure and delay in social decision making

Fadong Chen & Ian Krajbich

Supplementary Methods

Experiment. How to enforce time constraints in this experiment is an important problem. We chose to allow subjects to respond at any time, to avoid missing observations. However, if the time-constraint was disobeyed, we imposed a probabilistic penalty such that a dictator's decision was only implemented with a probability of 10%. In the other 90% of cases, the dictator earned 0 and the receiver earned the lower of the two amounts that she could earn in the current game. We chose this scheme relying on IIA (independence of irrelevant alternatives) to ensure that subjects would not choose a different option outside of the time constraints and trying to minimize the chance that subjects would prefer the penalized outcome (i.e. worst possible outcome for self, worst available outcome for the other). Subjects were notified with a warning message if they were too slow under time pressure or too fast under time delay (Supplementary Figure 1).

To make sure subjects understood their task, we provided a set of control questions at the beginning of each of the first three parts. In the experiment, we randomly displayed the selfish option and the pro-social option on the left and right sides of the display. We also randomized the display positions (upper or lower) of the dictator's payoffs and the receiver's payoffs across subjects. Subjects made decisions by pressing the "F" or "J" keys to select the left or right option, respectively. They saw a waiting screen after each decision and were required to press "Spacebar" to advance to the next trial. At the end of the experiment, we randomly selected one trial for each group and paid them according to one of their decisions (randomly selected). We programed the experiment using z-Tree[1] and recruited subjects via ORSEE[2]. The experimental instructions are as follows.

General Instructions

Welcome to this experimental study. If you read the following instructions carefully, you can - depending on your decisions - earn money in addition to the show-up fee of 10 dollars. Therefore, it is important that you read these instructions carefully.

During today's study you are not allowed to communicate with other participants. We also ask you to turn off your cell phone and put it away. If you have a question, please raise your hand and one of the experimenters will come by to answer it quietly.

There are four parts to the study. The instructions for each part will appear once you have finished the previous part.

In the instructions we do not speak of dollar, but points. The points you earn during the study will be converted to dollars at the following rate:

1 Point = 10 Cents

On the following screens you will receive detailed instructions for the study. When you finish reading the instructions, there will be comprehension questions to ensure that you understand how everything works.

Next Page

Today's Study

In today's study you will be deciding how to distribute money between yourself and another participant in the room. In each round there will be two available options. You simply need to pick one. These decisions have real consequences.

At the end of the study we will first randomly pair everyone into groups of two. Then we will randomly determine who in each group will be the decision-maker and who will be the non-decision-maker. Finally, we will randomly select one round for each group and pay the two group members according to the decision-maker's choice in that round.

In other words, there is a 50% chance that you will be the decision-maker and that one of your decisions will determine how much money you and another participant in the room earn today.

Previous Page

Next Page

Example round

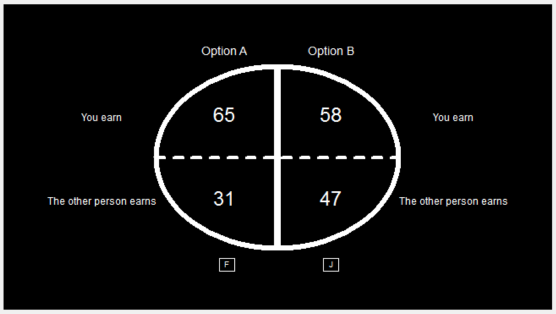

This is an example of the kind of decision you will be making in today's study. There are two possible options: the left option (Option A) and the right option (Option B). In Option A you get 65 points while the other person gets 31 points. In Option B you get 58 points while the other person gets 47 points. To choose Option A you would press the "F" key and to choose Option B you would press the "J" key. These keys are also indicated below each option, in case you forget.

Previous Page | Next Page

Between each round there will be a page that simply has a cross at the center of the screen. On this page, press the "Spacebar" to continue to the next round.

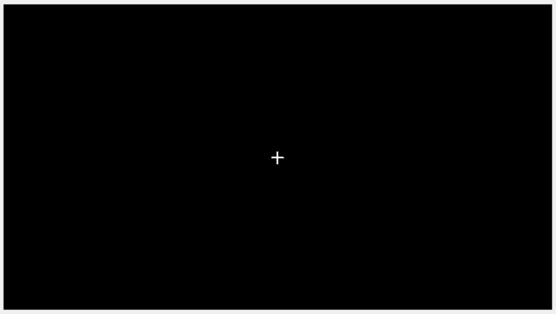

Previous Page | Continue

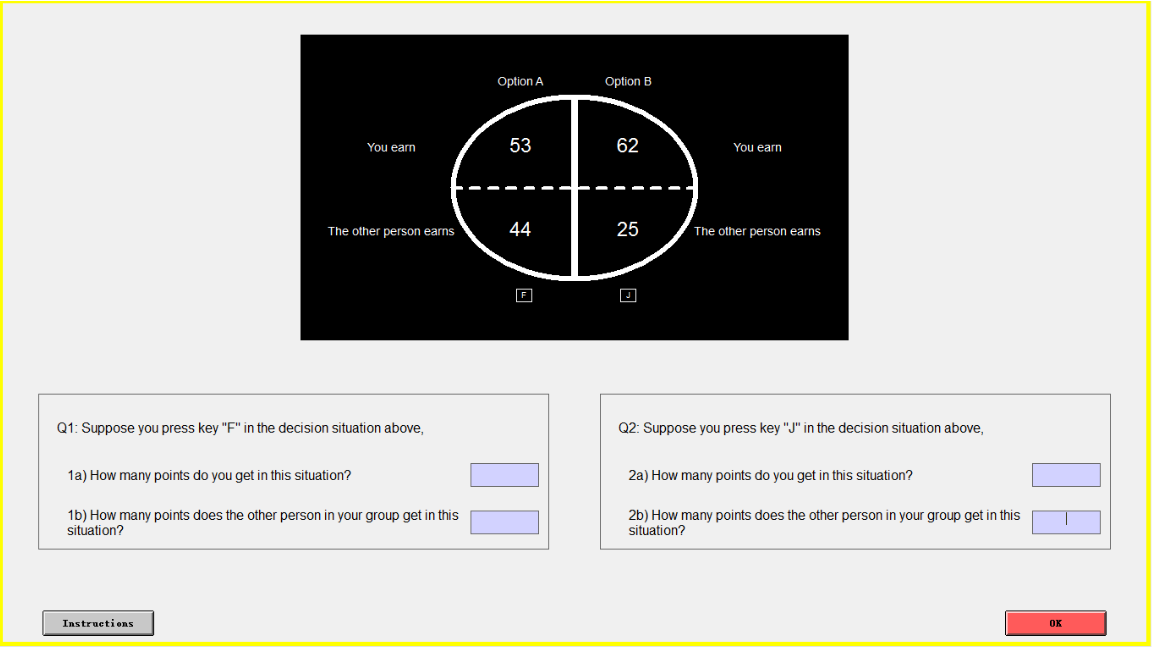
Option A
Option B
You earn
53
62
You earn
The other person earns
44
25
The other person earns
F
J
Q1: Suppose you press key "F" in the decision situation above,
1a) How many points do you get in this situation?
1b) How many points does the other person in your group get in this situation?
Q2: Suppose you press key "J" in the decision situation above,
2a) How many points do you get in this situation?
2b) How many points does the other person in your group get in this situation?
Instructions
OK

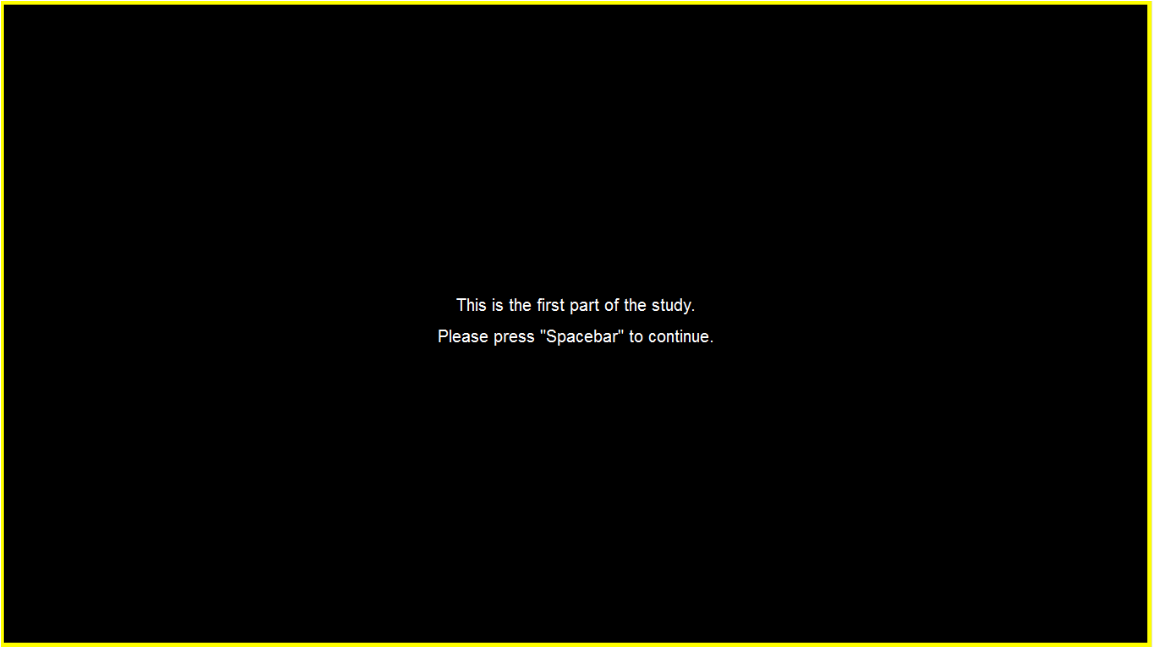
This is the first part of the study.
Please press "Spacebar" to continue.

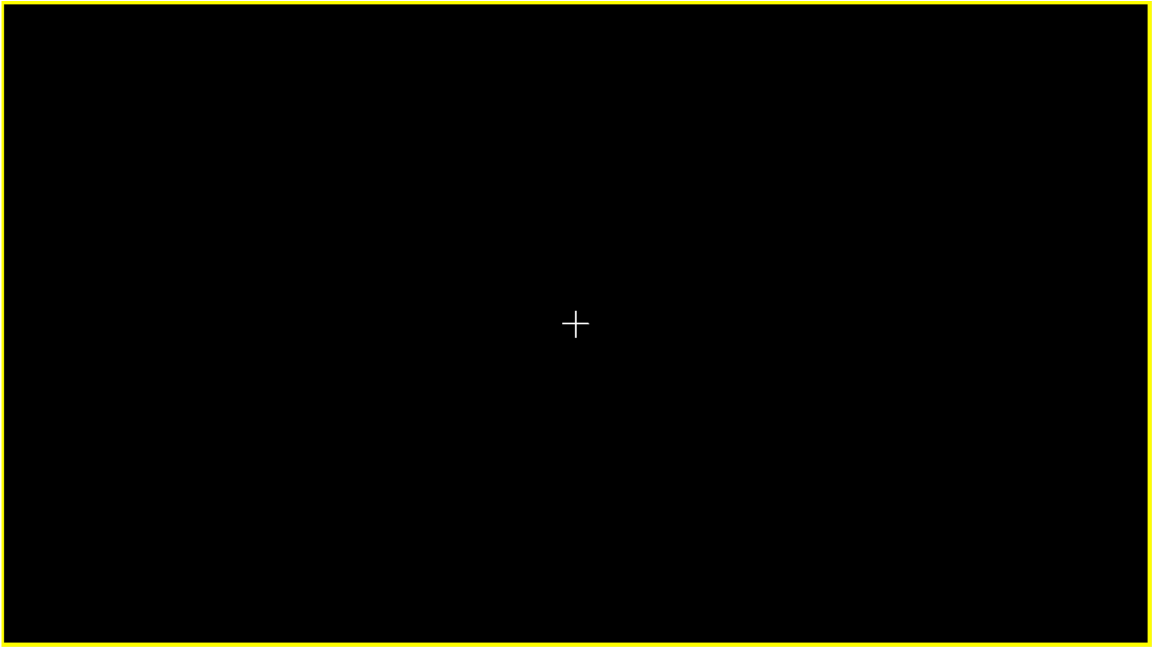

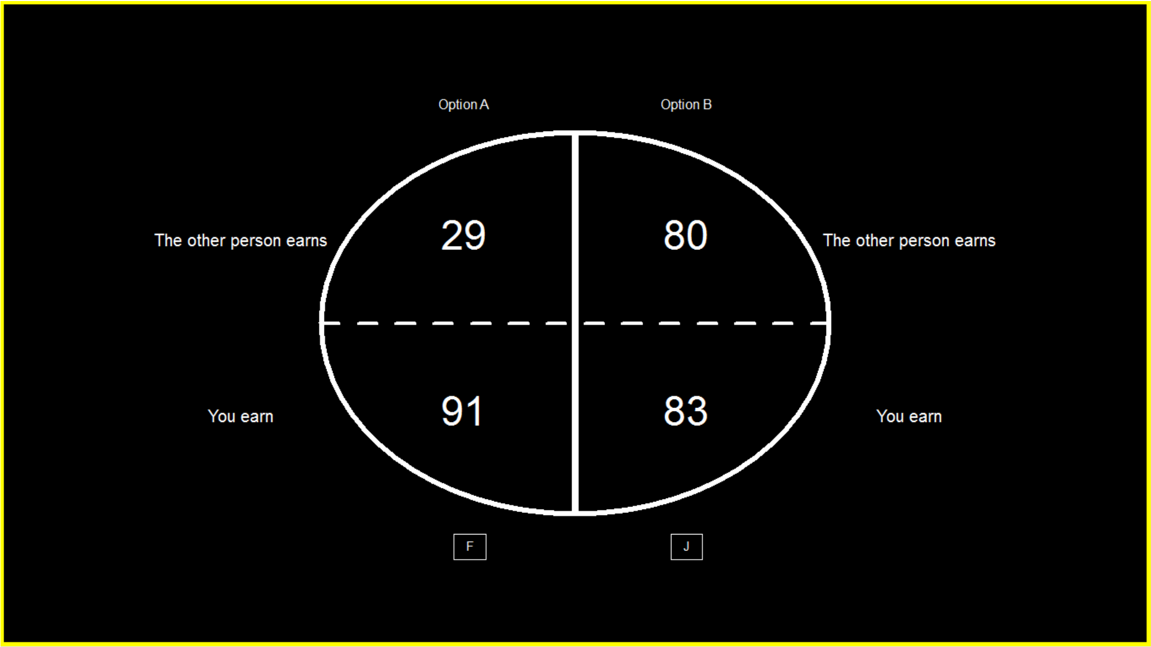
Option A
Option B
The other person earns
29
80
The other person earns
You earn
91
83
You earn
F
J

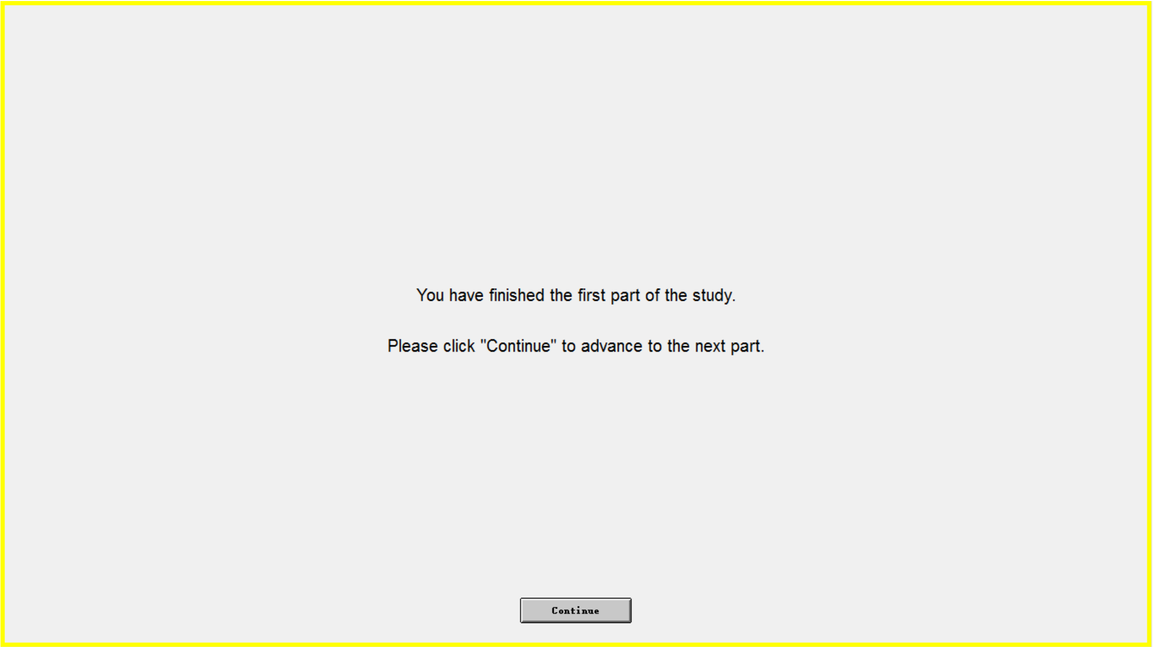
You have finished the first part of the study.
Please click "Continue" to advance to the next part.
Continue

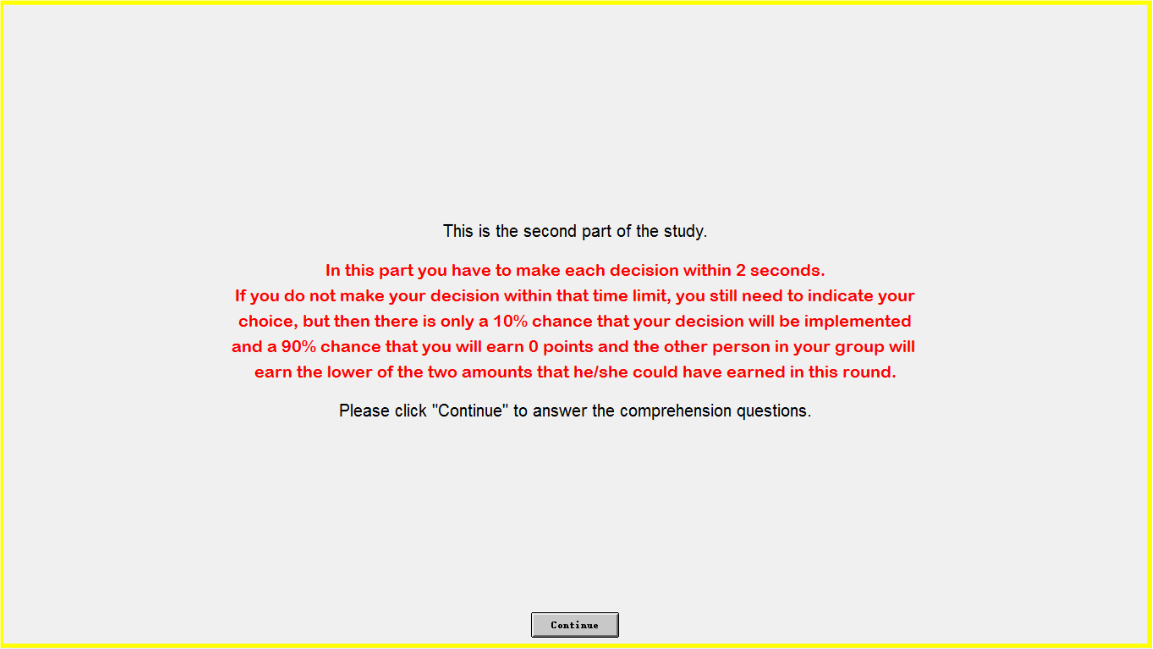
This is the second part of the study.
In this part you have to make each decision within 2 seconds.
If you do not make your decision within that time limit, you still need to indicate your choice, but then there is only a 10% chance that your decision will be implemented and a 90% chance that you will earn 0 points and the other person in your group will earn the lower of the two amounts that he/she could have earned in this round.
Please click "Continue" to answer the comprehension questions.
Continue

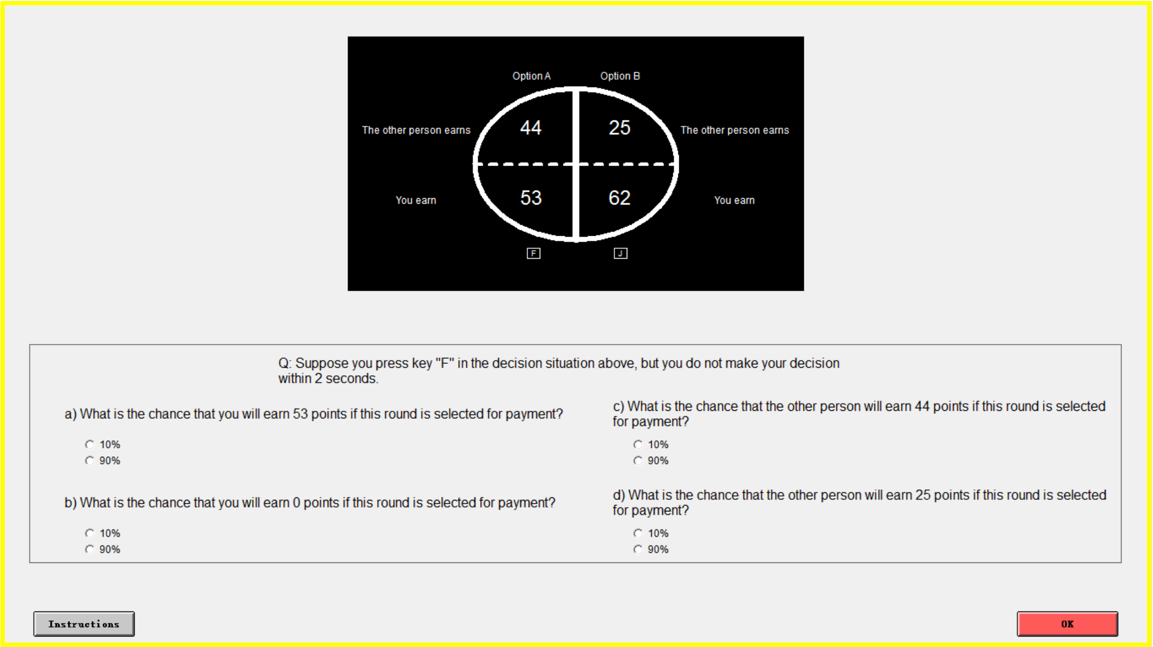

Option A
Option B
The other person earns
44
25
The other person earns
You earn
53
62
You earn
F
J
Q: Suppose you press key "F" in the decision situation above, but you do not make your decision within 2 seconds.
a) What is the chance that you will earn 53 points if this round is selected for payment?
10%
90%
b) What is the chance that you will earn 0 points if this round is selected for payment?
10%
90%
c) What is the chance that the other person will earn 44 points if this round is selected for payment?
10%
90%
d) What is the chance that the other person will earn 25 points if this round is selected for payment?
10%
90%
Instructions
OK

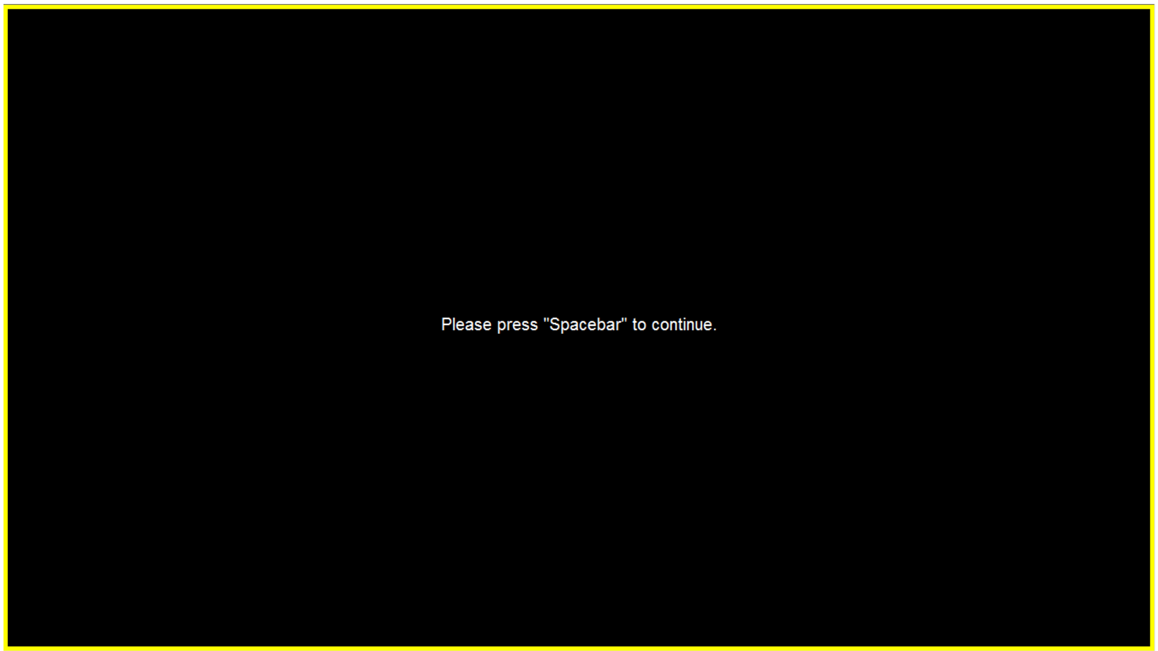

Please press "Spacebar" to continue.

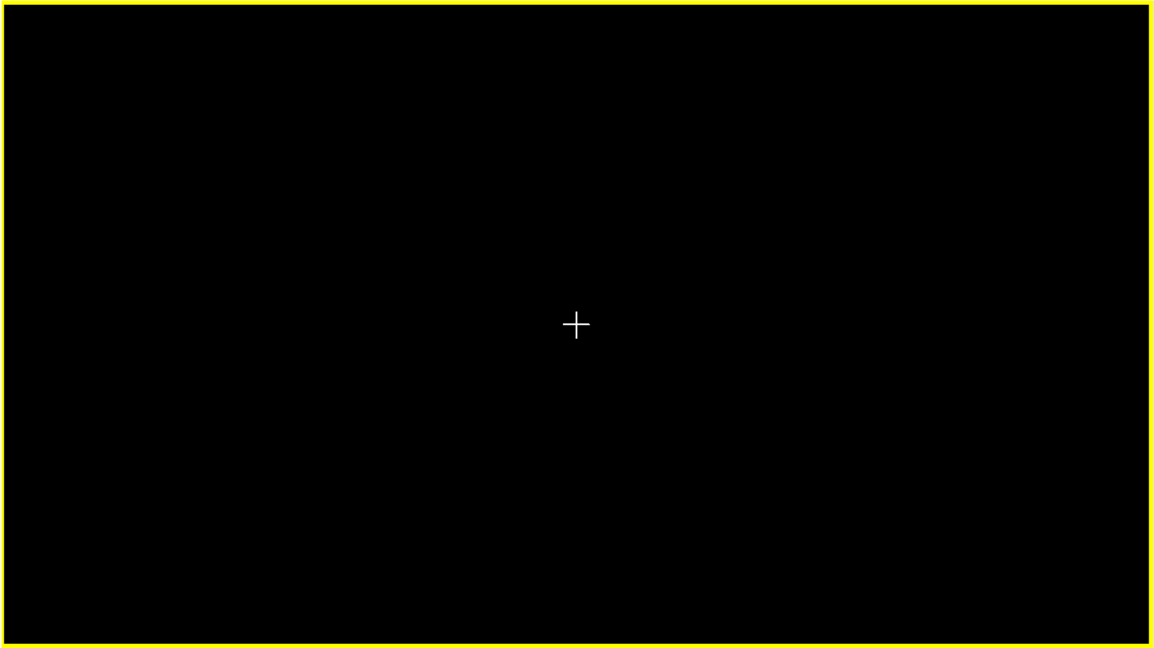

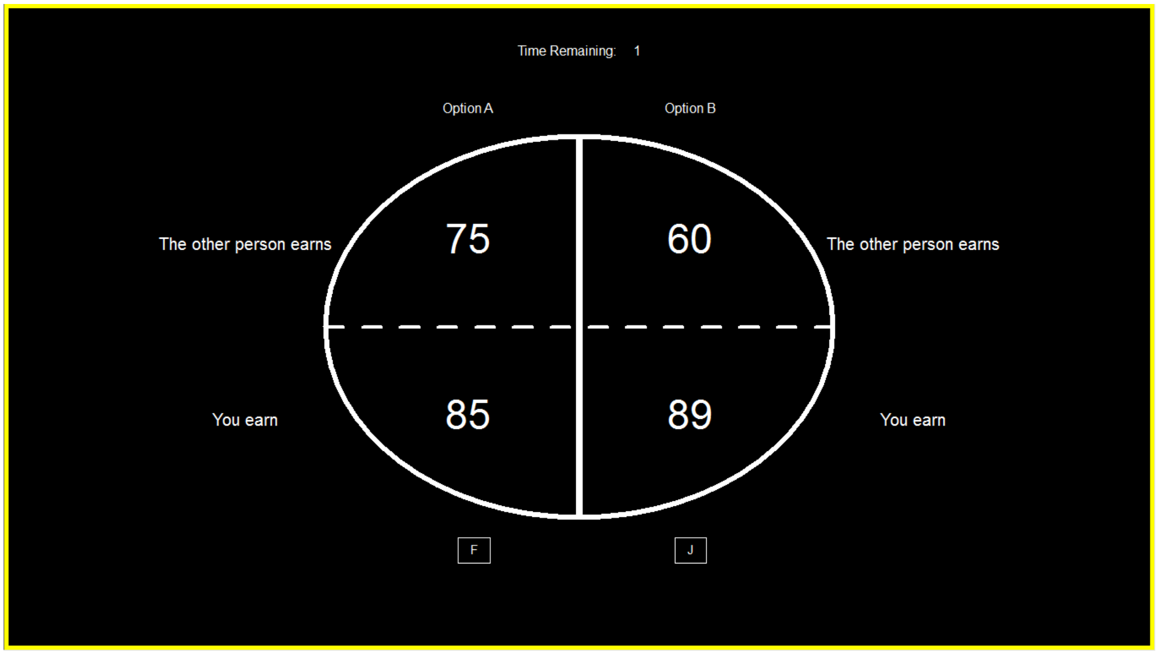
Time Remaining: 1
Option A
Option B
The other person earns
75
60
The other person earns
You earn
85
89
You earn
F
J

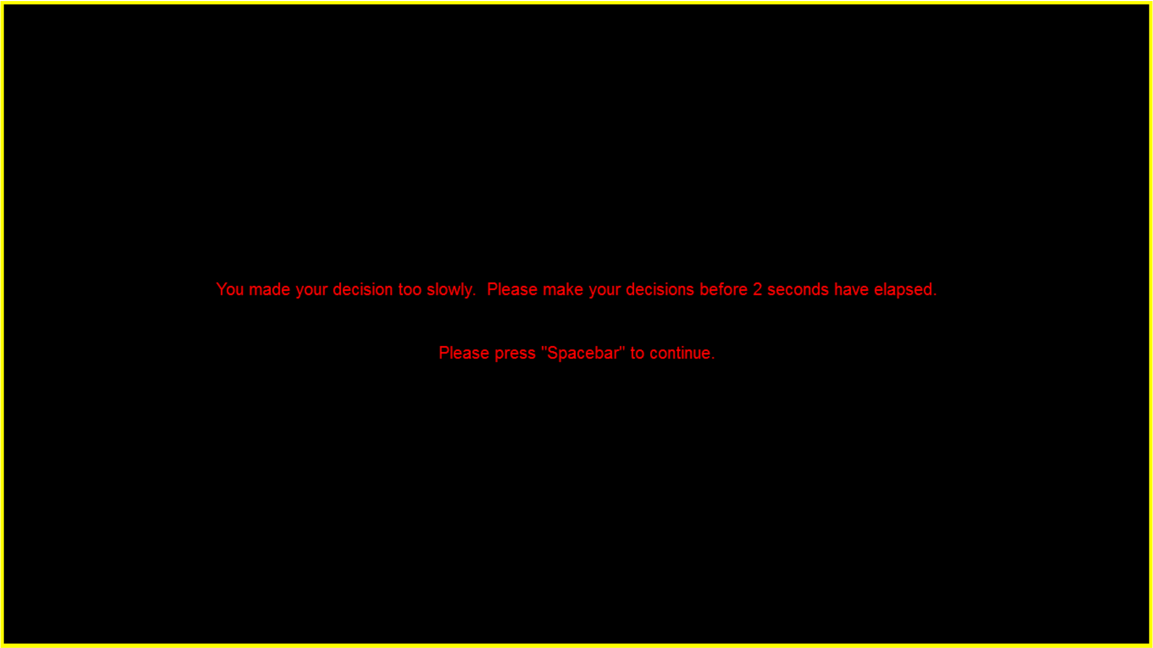
You made your decision too slowly. Please make your decisions before 2 seconds have elapsed.
Please press "Spacebar" to continue.

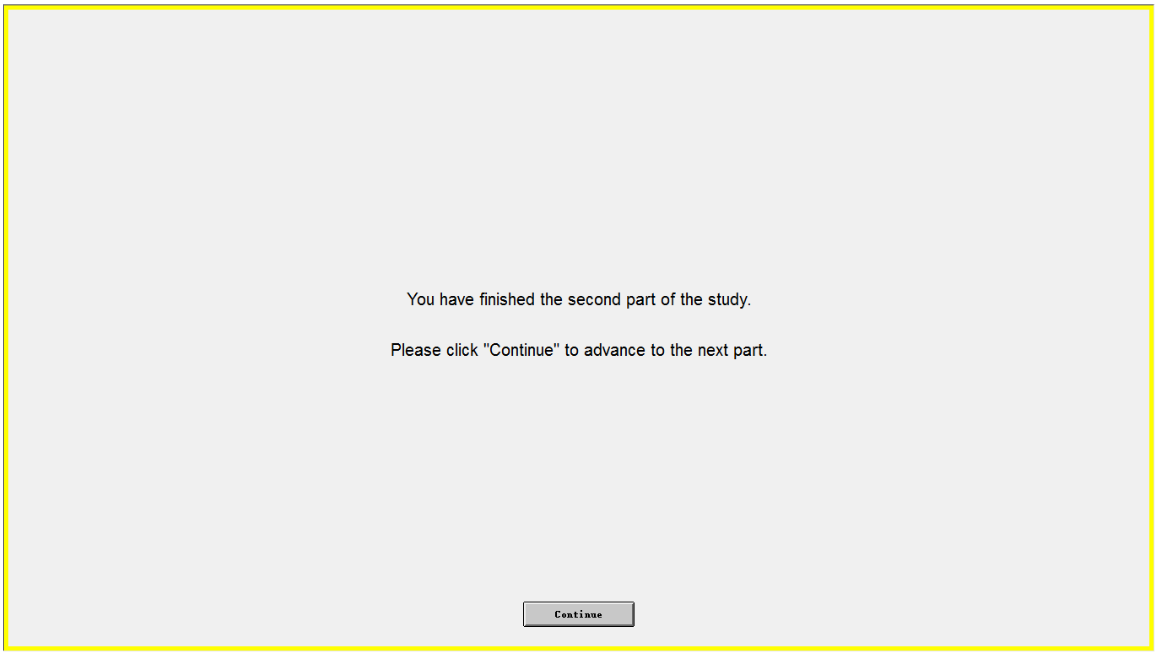
You have finished the second part of the study.
Please click "Continue" to advance to the next part.
Continue

This is the third part of the study.

In this part you must wait 10 seconds before making your decision.
If you do not wait for the full 10 seconds then there is only a 10% chance that your decision will be implemented and a 90% chance that you will earn 0 points and the other person in your group will earn the lower of the two amounts that he/she could have earned in this round. Also, please note that you cannot speed up this part of the study by choosing quickly because no matter when you make your choice, you will always have to wait 10 seconds.

Please click "Continue" to answer the comprehension questions.

Continue

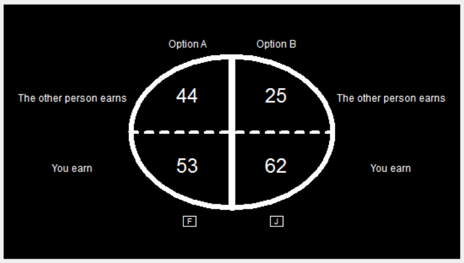

Q: Suppose you press the "F" key in the decision situation above, but you press the key before 10 seconds has elapsed.

a) What is the chance that you will earn 53 points if this round is selected for payment?

- 10%
- 90%

b) What is the chance that you will earn 0 points if this round is selected for payment?

- 10%
- 90%

c) What is the chance that the other person will earn 44 points if this round is selected for payment?

- 10%
- 90%

d) What is the chance that the other person will earn 25 points if this round is selected for payment?

- 10%
- 90%

Instructions

OK

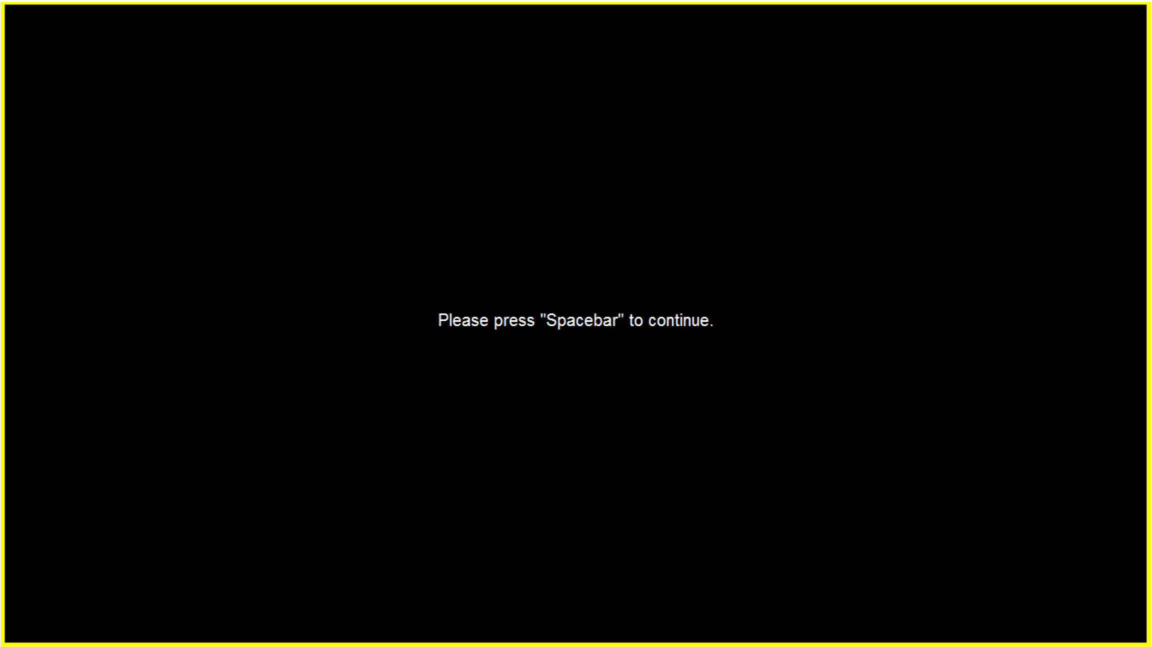
Please press "Spacebar" to continue.

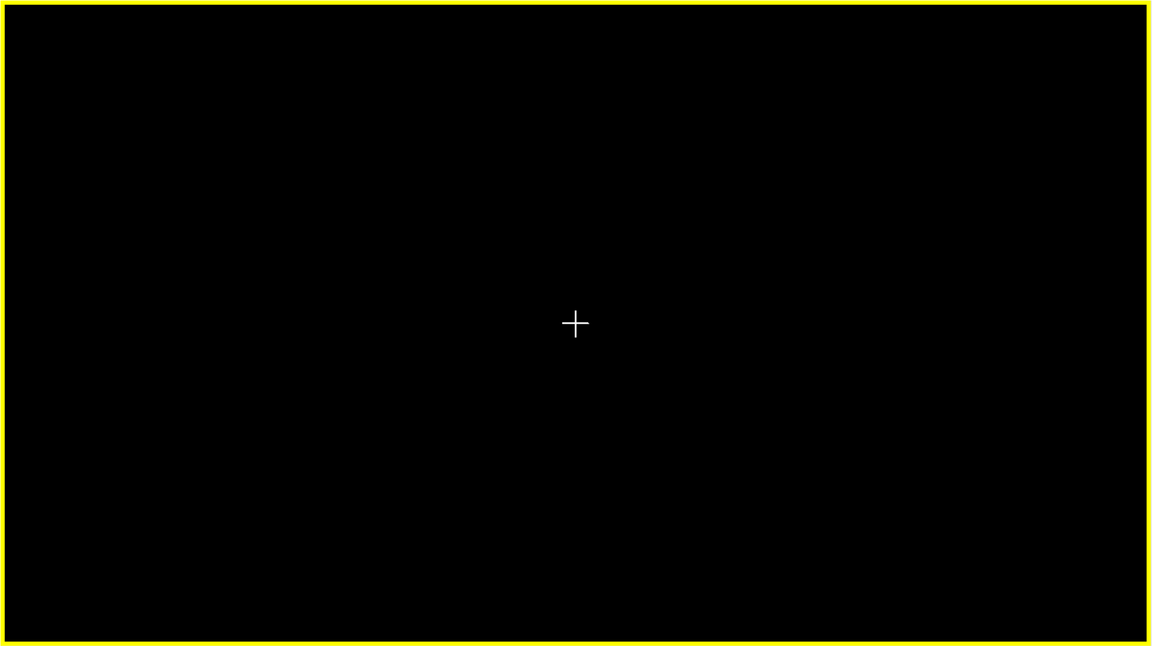

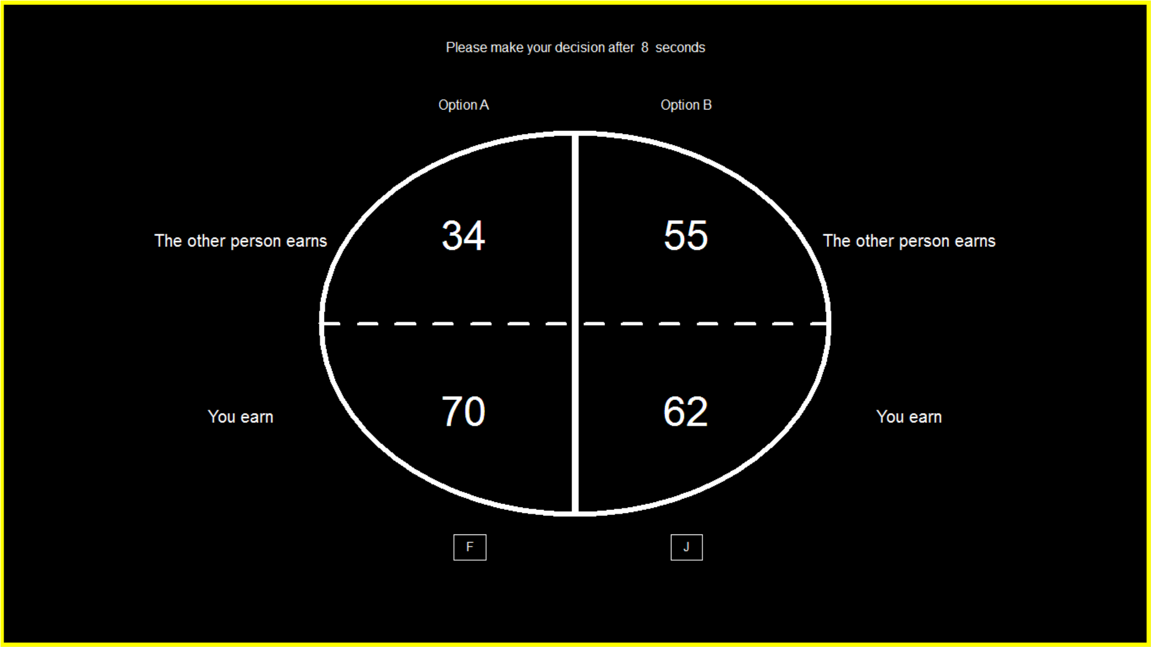
Please make your decision after 8 seconds
Option A
Option B
The other person earns
34
55
The other person earns
You earn
70
62
You earn
F
J

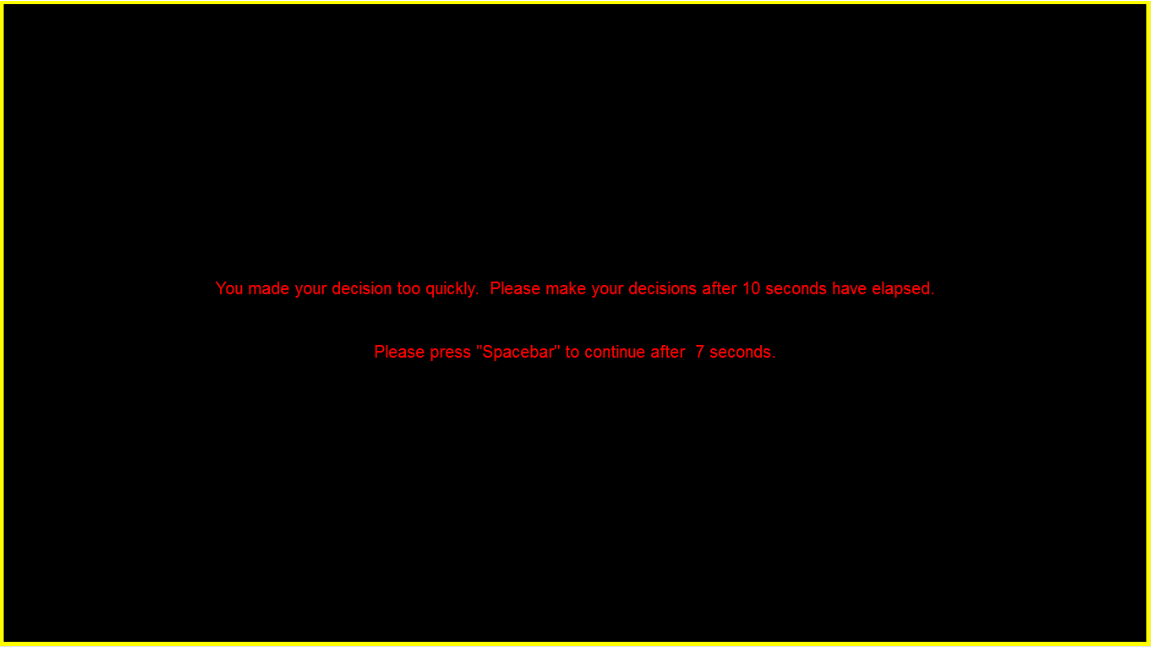
You made your decision too quickly. Please make your decisions after 10 seconds have elapsed.
Please press "Spacebar" to continue after 7 seconds.

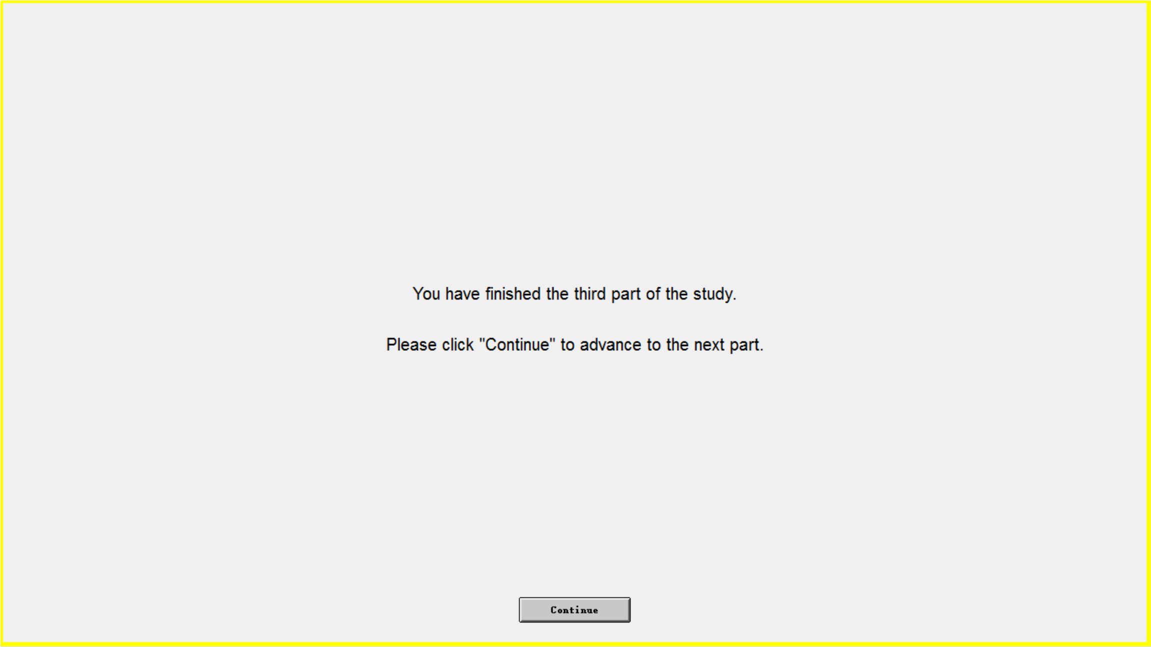
You have finished the third part of the study.
Please click "Continue" to advance to the next part.
Continue

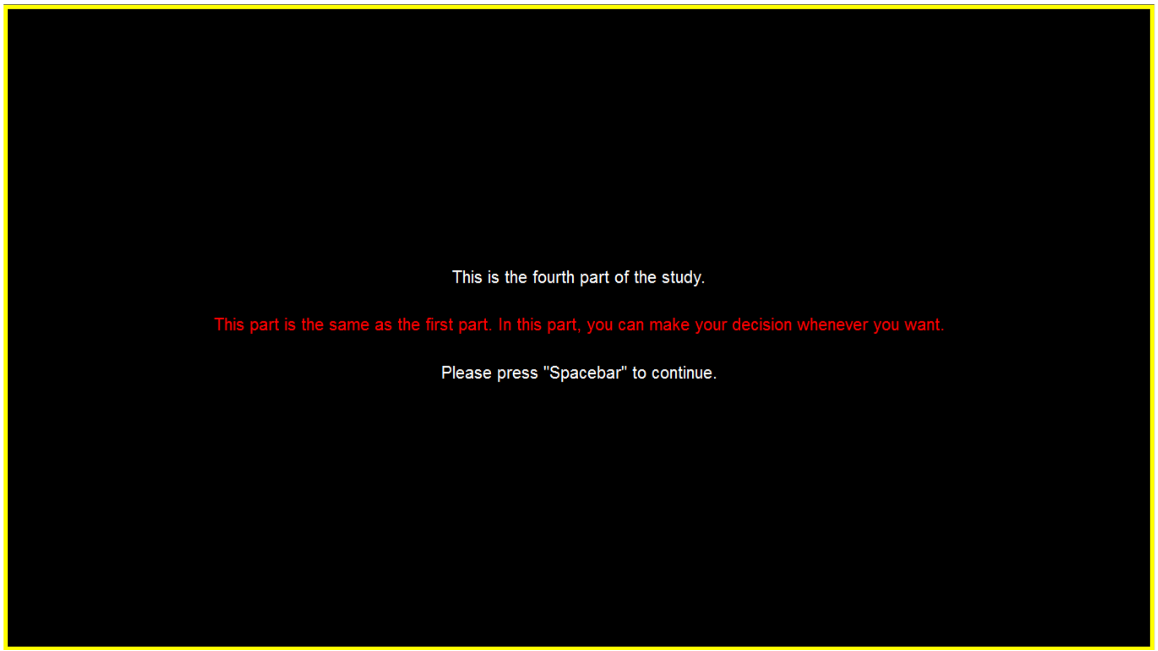
This is the fourth part of the study.
This part is the same as the first part. In this part, you can make your decision whenever you want.
Please press "Spacebar" to continue.

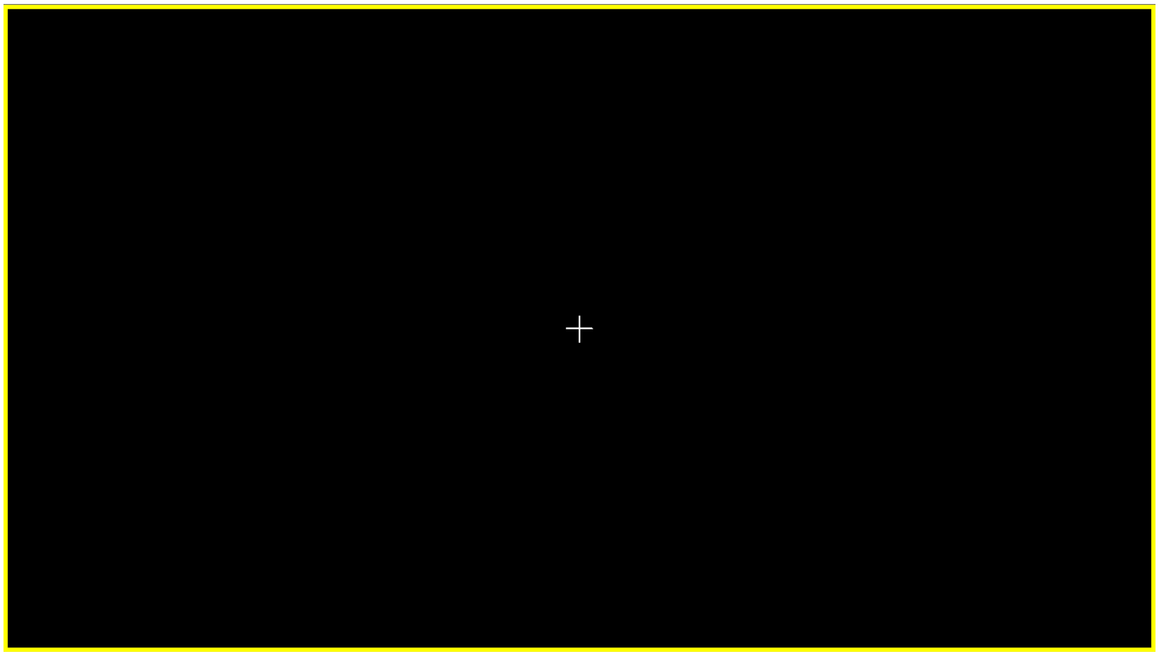

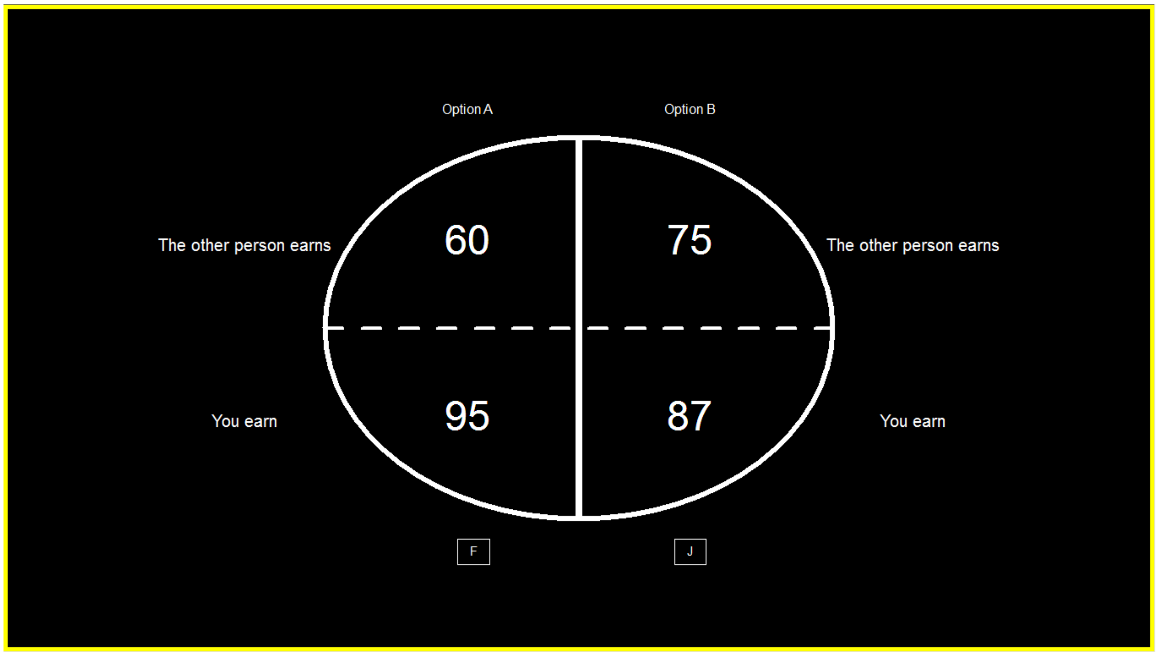
Option A
Option B
The other person earns
60
75
The other person earns
You earn
95
87
You earn
F
J

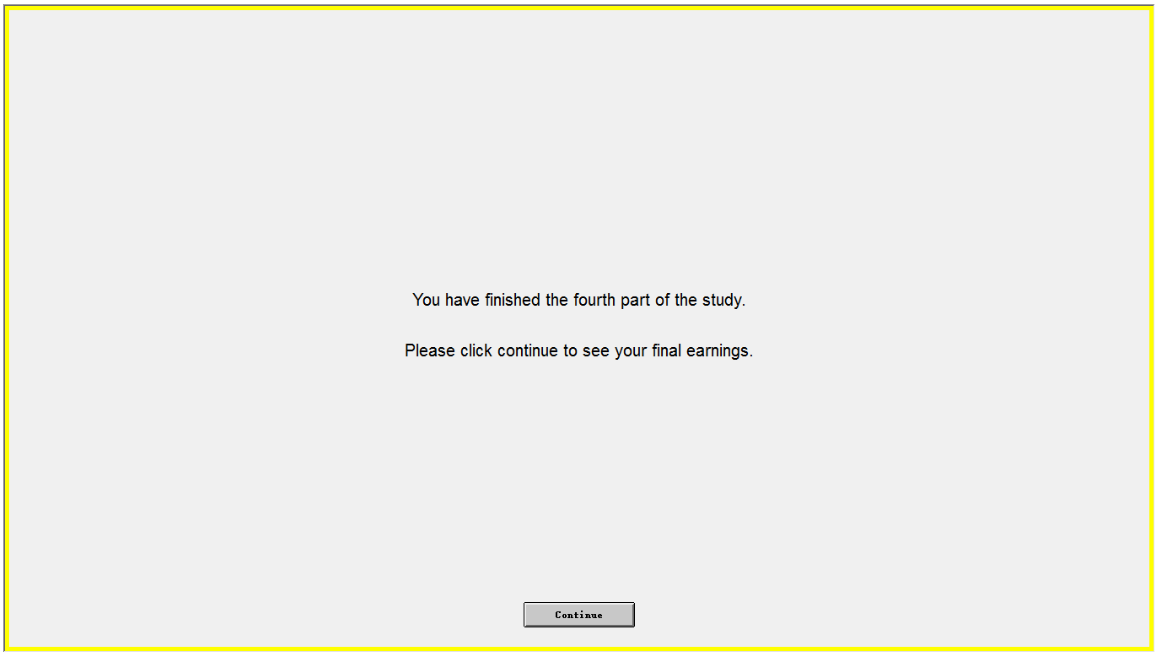
You have finished the fourth part of the study.
Please click continue to see your final earnings.
Continue

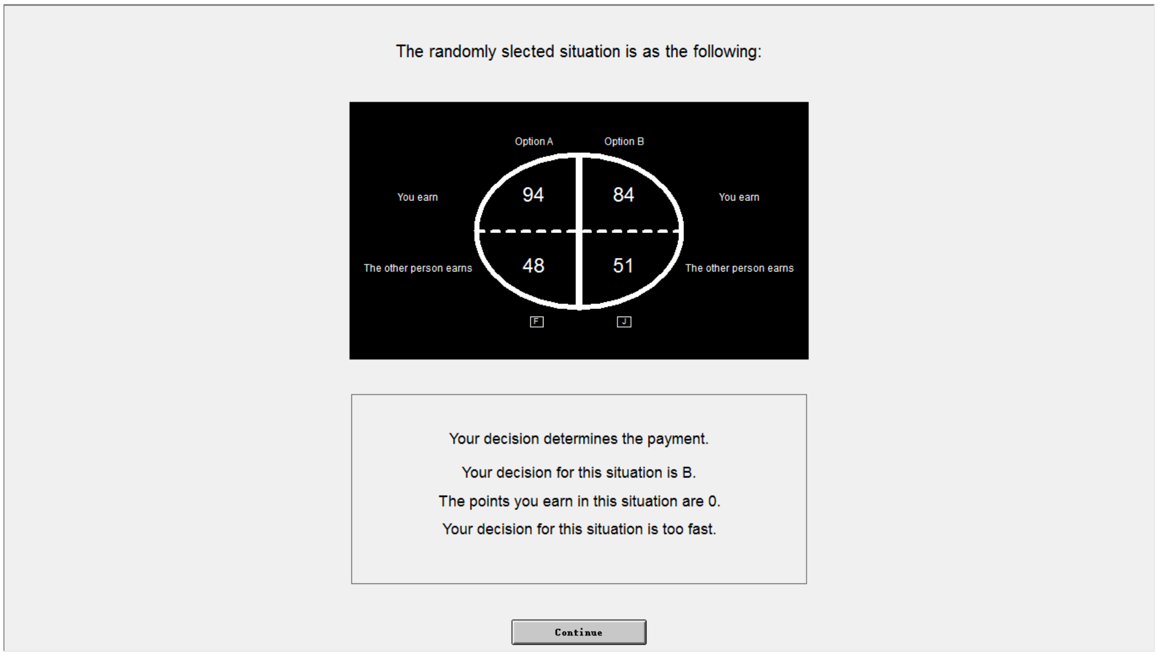
The randomly slected situation is as the following:
Option A
Option B
You earn
94
84
You earn
The other person earns
48
51
The other person earns
F
J
Your decision determines the payment.
Your decision for this situation is B.
The points you earn in this situation are 0.
Your decision for this situation is too fast.
Continue

Supplementary Notes

Supplementary Note 1. Consistent with prior work in this literature [3,4], our subjects generally did care about both players' payoffs. In the experiment, subjects chose the selfish option in 42.3% of all games in the time-free condition, they chose the selfish option in 50.7% of all games in the time-pressure condition, and they chose the selfish option in 40.3% of all games in the time-delay condition (Supplementary Figure 9). The median RTs for decisions in the time-free, time-pressure, and time-delay conditions were 2.156 s (sd = 2.797), 0.937 s (sd = 0.393), and 11.470 s (sd = 1.642), respectively (Supplementary Figure 5). Subjects violated the time constraints in 1.6% of time-pressure trials and 1.0% of time-delay trials.

Supplementary Note 2. In addition to using the median indifference β, we also considered three other cutoffs to delineate between selfish and pro-social subjects. One is the median subject-level β_{f}. The median subject-level β_{f} in our experiment was 0.288. Using this cutoff, 73% of the selfish subjects chose the selfish option on the majority of trials, while 100% of the pro-social subjects chose the pro-social option on the majority of trials. With this cutoff, the pro-social subjects (n=51) become more pro-social under time pressure (P = 0.051, two-sided Wilcoxon signed-rank test, since β is not normally distributed), while selfish subjects (n=51) become marginally more selfish under time pressure (P = 0.221). Similarly, pro-social subjects become less pro-social under time delay (P = 0.054), while selfish subjects become less selfish under time delay (P = 0.001).

The second cutoff is the average β reported in Fehr & Schmidt (1999). The β reported in Fehr & Schmidt (1999) was 0.315. With this cutoff, 67% of the selfish subjects chose the selfish option on the majority of trials; while 100% of the pro-social subjects chose the pro-social option on the majority of trials. With this cutoff, pro-social subjects (n=47) become more pro-social under time pressure (P = 0.024, two-sided Wilcoxon signed-rank test), while selfish subjects (n=55) become marginally more selfish under time pressure (P = 0.098). Similarly, pro-social subjects become less pro-social under time delay (P = 0.052), while selfish subjects become less selfish under time delay (P = 0.001).

The third cutoff is the point at which $\beta_{\mathrm{p}} = \beta_{\mathrm{f}}$ (0.104) in the biased DDM simulations in Supplementary Figure 4c. With this cutoff, 97% of the selfish subjects chose the selfish option on the majority of trials; while 89% of the pro-social subjects chose the pro-social option on the majority of trials. With this cutoff, pro-social subjects (n=72) become more pro-social under time pressure (P = 0.044, two-sided Wilcoxon signed-rank test), while selfish subjects (n=30) become marginally more

selfish under time pressure (P = 0.077). Similarly, pro-social subjects become less pro-social (but not significantly so) under time delay (P = 0.278), while selfish subjects become less selfish under time delay (P = 0.005).

Supplementary Note 3. To ensure that the biases in starting point were not driven by the selfish (pro-social) behavior of subjects, we simulated a selfish subject with a starting point 0.5 and a drift rate of 0.6. In these simulations, 85.4% of all decisions were selfish decisions. Then we estimated the biased DDM based on the simulated data. We recovered a starting point of 0.484. Therefore, selfish (pro-social) preferences do not appear to produce starting point biases.

To check whether the biases in starting points might have been caused by unaccounted for variability across subjects, we simulated ten selfish subjects with a starting point 0.5 and drift rates 0.1 to 1 in step of 0.1. In the simulations, 79.7% of all decisions were selfish decisions. We then estimate the biased DDM at the group level based on the simulated data. We recovered a starting point of 0.502. Therefore, drift rate variability across subjects does not appear to produce starting-point biases.

Supplementary Figures

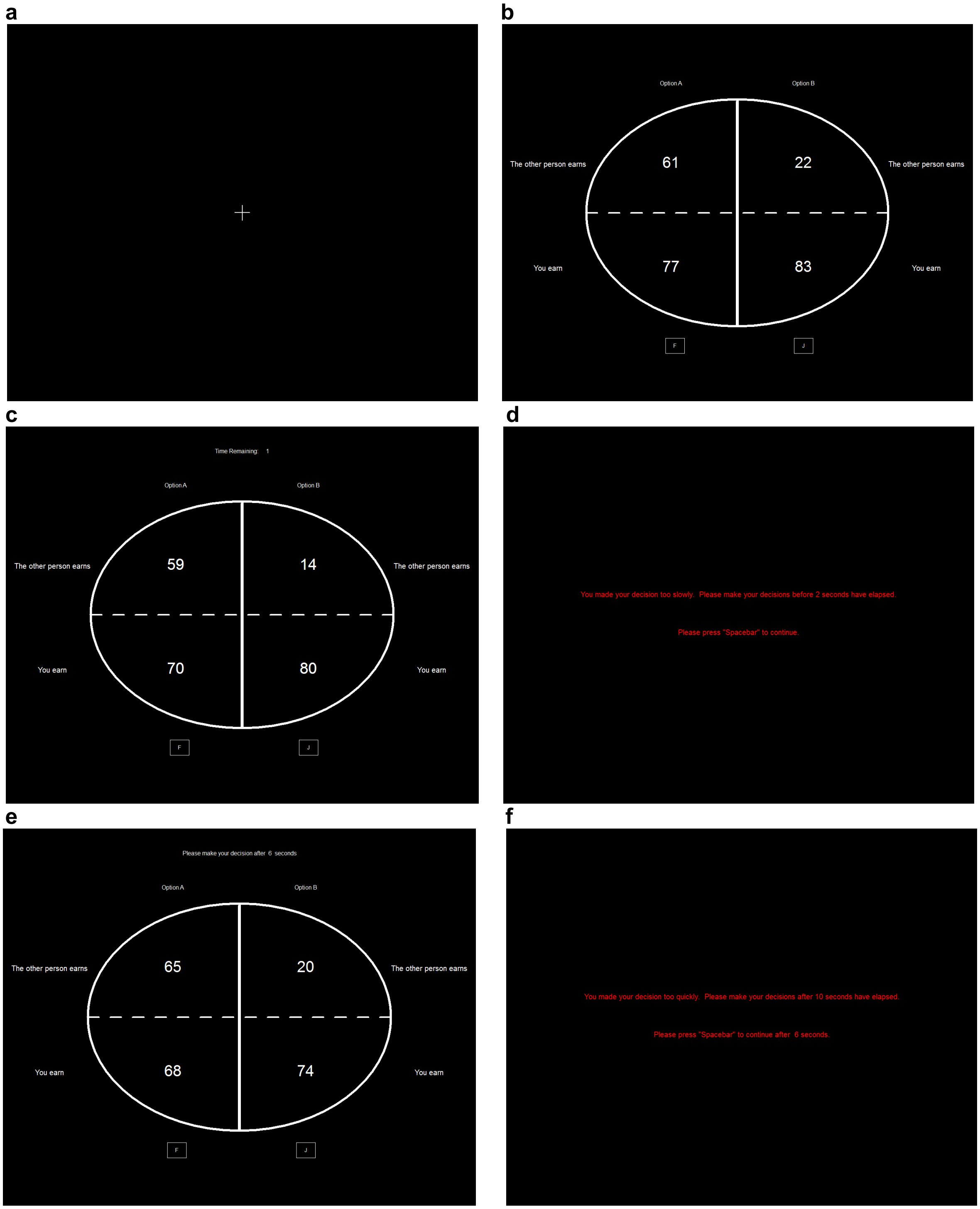

Supplementary Figure 1. Choice screens in the experiment. (**a**) Each trial began with a fixation screen. Subjects pressed the 'Spacebar' to advance to the decision screen. (**b**) Choice screen in the time-free condition. (**c**) Choice screen in the time-pressure condition. (**d**) The warning message for subjects who decided too slowly in the time-pressure condition. (**e**) Choice screen in the time-delay condition. (**f**) The warning message for subjects who decided too quickly in the time-delay condition.

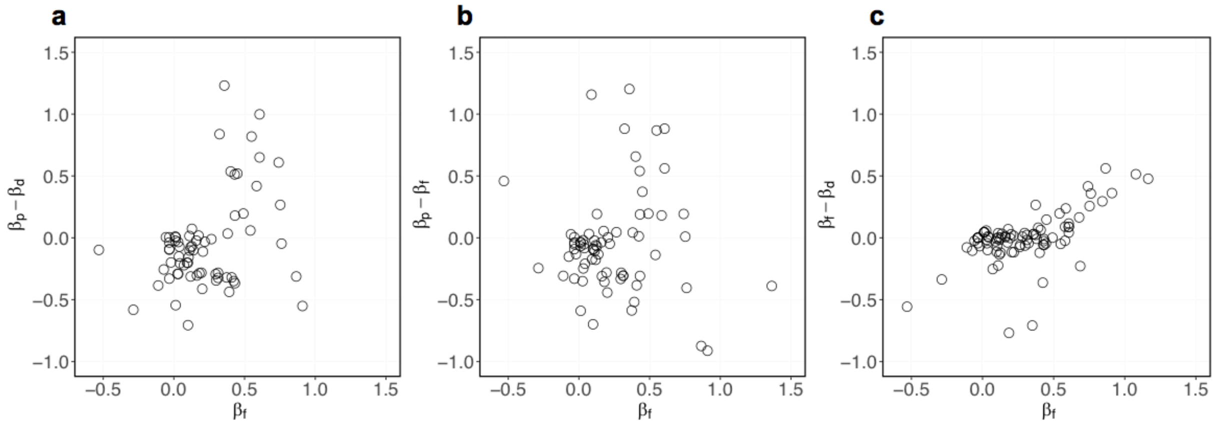

Supplementary Figure 2. Correlations between $\boldsymbol{\beta}_{\mathrm{f}}$ and $\boldsymbol{\beta}$ change across time conditions. (**a**) plots β_{f} vs. $\beta_{\mathrm{p}} - \beta_{\mathrm{d}}$, (**b**) plots β_{f} vs. $\beta_{\mathrm{p}} - \beta_{\mathrm{f}}$, and (**c**) plots β_{f} vs. $\beta_{\mathrm{f}} - \beta_{\mathrm{d}}$. For display purpose, (**a**) includes 69 subjects whose β_{f}, β_{p}, and β_{d} are between -1 and 2, (**b**) includes 71 subjects whose β_{f} and β_{p} are between -1 and 2, and (**c**) includes 88 subjects whose β_{f} and β_{d} are between -1 and 2.

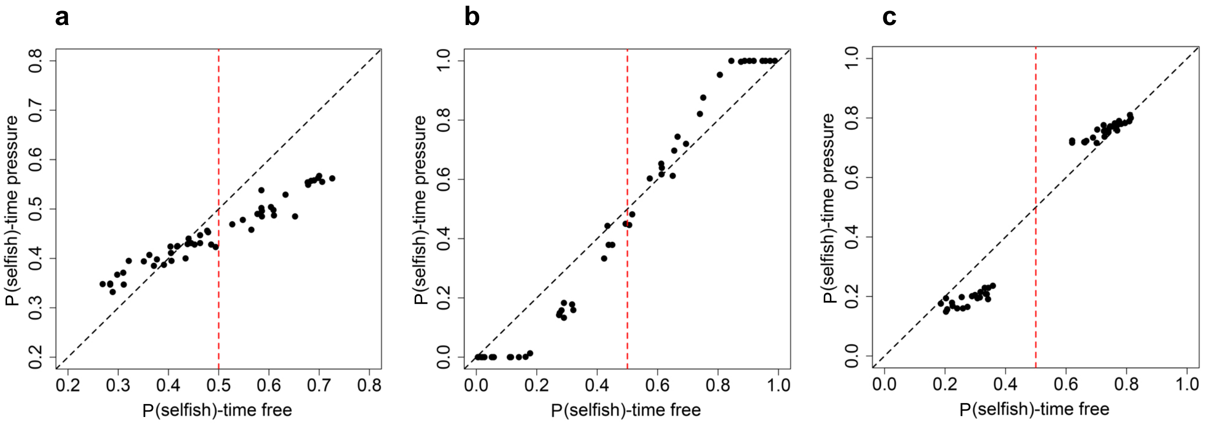

Supplementary Figure 3. Simulations of the DDM with exponentially collapsing thresholds. In the simulations, the threshold collapses with $a(t) = e^{-rt}$, r=0.005 ms^{-1}. (**a**) the simple DDM with no starting point biases (unbiased DDM), (**b**) the DDM with starting point biases proportional to drift rate (biased DDM), and (**c**) the DDM with a fixed starting point bias of +/- 0.25 (biased DDM). Each dot represents one simulated subject. Note that in (**a**) the dots fall between the diagonal (black dashed line) and the horizontal midline, indicating that under time pressure these subjects' choices move towards chance, while in (**b**) and (**c**) the dots fall between the diagonal and the vertical midline (red dashed line), indicating that under time pressure these subjects' choices become more extreme. Only this latter pattern is consistent with the results seen in Fig. 2. The effects of collapsing boundaries are very similar to the effects of tighter boundaries: starting points exert a greater effect on choice with them present.

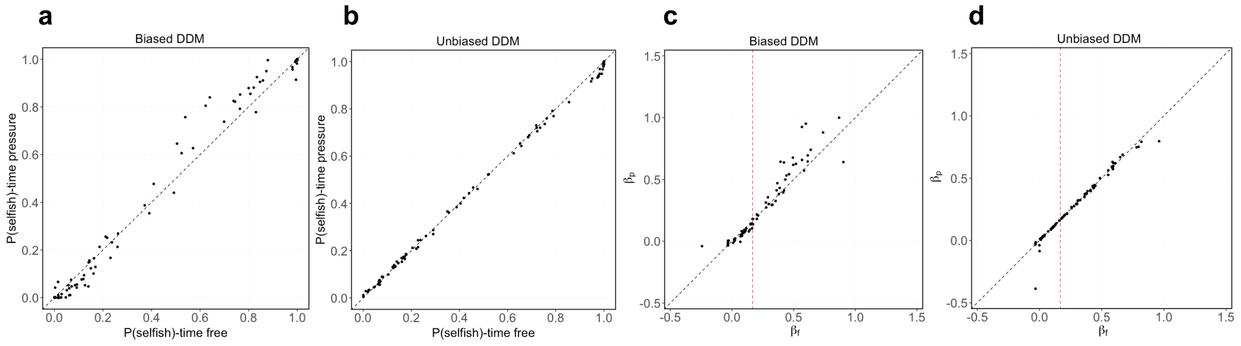

Supplementary Figure 4. DDM simulations using the parameters estimated form the experimental data. (**a**) the simulated probability of choosing the selfish option in the time-free and time-pressure conditions in the biased DDM (DDM with biased starting points) simulations, (**b**) the simulated probability in the unbiased DDM (DDM without starting point biases) simulations, (**c**) the advantageous inequality aversion (β) estimated from the simulated data in the biased DDM simulations (data for plot panel **a**), and (**d**) the advantageous inequality aversion (β) estimated from the simulated data in the unbiased DDM simulations (data for plot panel **b**). We carried out the simulations using the parameters of the 86 subjects whose $\beta_{\mathrm{f}} \in [-0.5, 1]$. We used β_{f} to calculate the utility difference (UD) between choice options and let the drift rate (dr) be proportional to UD, that is, $dr = 0.2 * UD$. We used the threshold (a) estimated from the experimental data in the time-free simulations, and we used $0.5a$ in the time-pressure simulations. Each dot represents one subject. The black dotted line is the identity line. Note that in (**a**) subjects' choices become more extreme under time pressure in the biased DDM simulations (two-sided Wilcoxon signed rank tests, P = 0.005 for selfish subjects and P = 0.011 for pro-social subjects (split by the median indifference β)), in (**b**) subjects' choices move towards chance under time pressure in the unbiased DDM simulations ($P < 10^{-5}$ for selfish subjects and P = 0.025 for pro-social subjects), in (**c**) subjects to the left of the vertical red line (split by the median indifference β, selfish subjects) are consistently shifted downwards while those to the right (pro-social subjects) are consistently shifted upwards in the biased DDM simulations (P = 0.033 for selfish subjects and P < 0.001 for pro-social subjects), and in (**d**) subjects in the unbiased DDM simulations do not show that pattern as in (**c**) and Fig. 2 (P = 0.774 for selfish subjects and P = 0.297 for prosocial subjects). Therefore, only the pattern produced by the biased DDM simulations (**a** and **c**) is consistent with the results observed in the experimental data (Fig. 2).

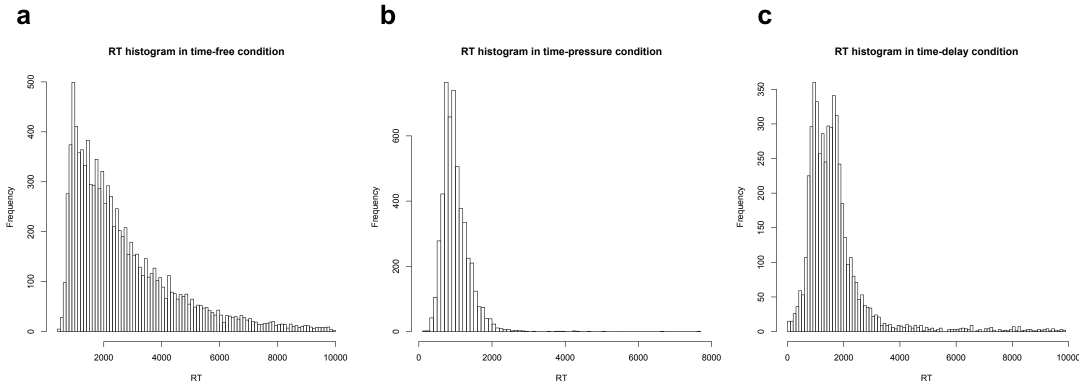

Supplementary Figure 5. Response time (RT) distributions for the three time conditions. (**a**) RT distribution for the time-free condition, (**b**) RT distribution for the time-pressure condition, and (**c**) RT distribution for the time-delay (after subtracting 10 s) condition. We see a standard skewed RT distribution in the time-free condition, while we see roughly Normal distributions under time pressure and time delay.

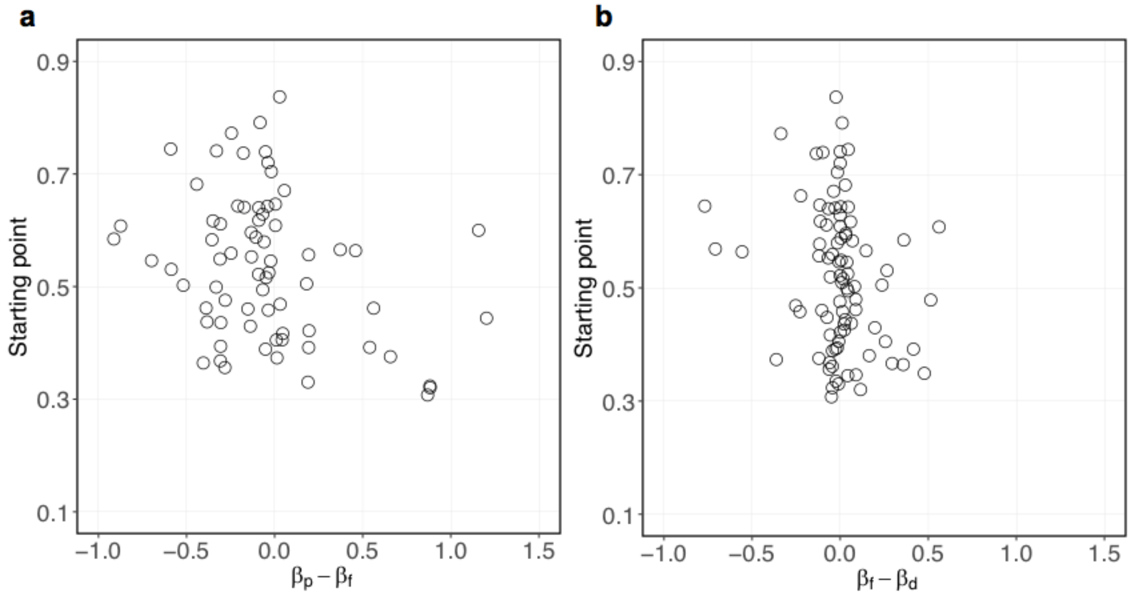

Supplementary Figure 6. Correlations between the starting point and β change across time conditions. (**a**) plots the correlation between the starting point and $\beta_p - \beta_f$, and (**b**) plots the correlation between the starting point and $\beta_f - \beta_d$. For display purpose, (**a**) includes 71 subjects whose β_p and β_f is between -1 and 2, and (**b**) includes 88 subjects whose β_f and β_d is between -1 and 2.

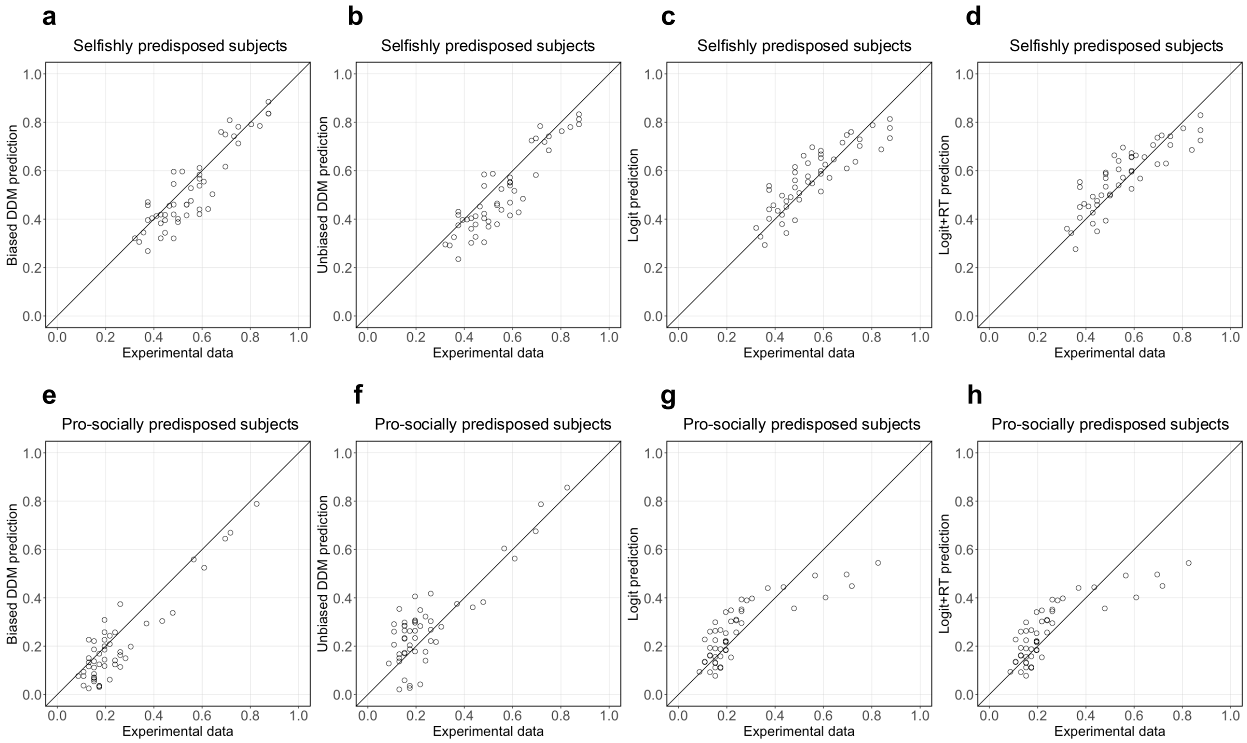

Supplementary Figure 7. Comparisons of the predictions by different models with the experimental data. Here we use Games 1-50 to predict decisions in Games 51-100. Each dot represents one game. X-axis is the probability of selfish choice in the experimental data, and Y-axis is the predicted probability of selfish choice by the biased DDM, the unbiased DDM, the Logit, and the Logit+RT. (**a**) The predicted probability by the biased DDM for selfishly predisposed subjects. (**b**) The predicted probability by the unbiased DDM for selfishly predisposed subjects. (**c**) The predicted probability by the Logit for selfishly predisposed subjects. (**d**) The predicted probability by the Logit+RT for selfishly predisposed subjects. (**e**) The predicted probability by the biased DDM for pro-socially predisposed subjects. (**f**) The predicted probability by the unbiased DDM for pro-socially predisposed subjects. (**g**) The predicted probability by the Logit for pro-socially predisposed subjects. (**h**) The predicted probability by the Logit+RT for pro-socially predisposed subjects.

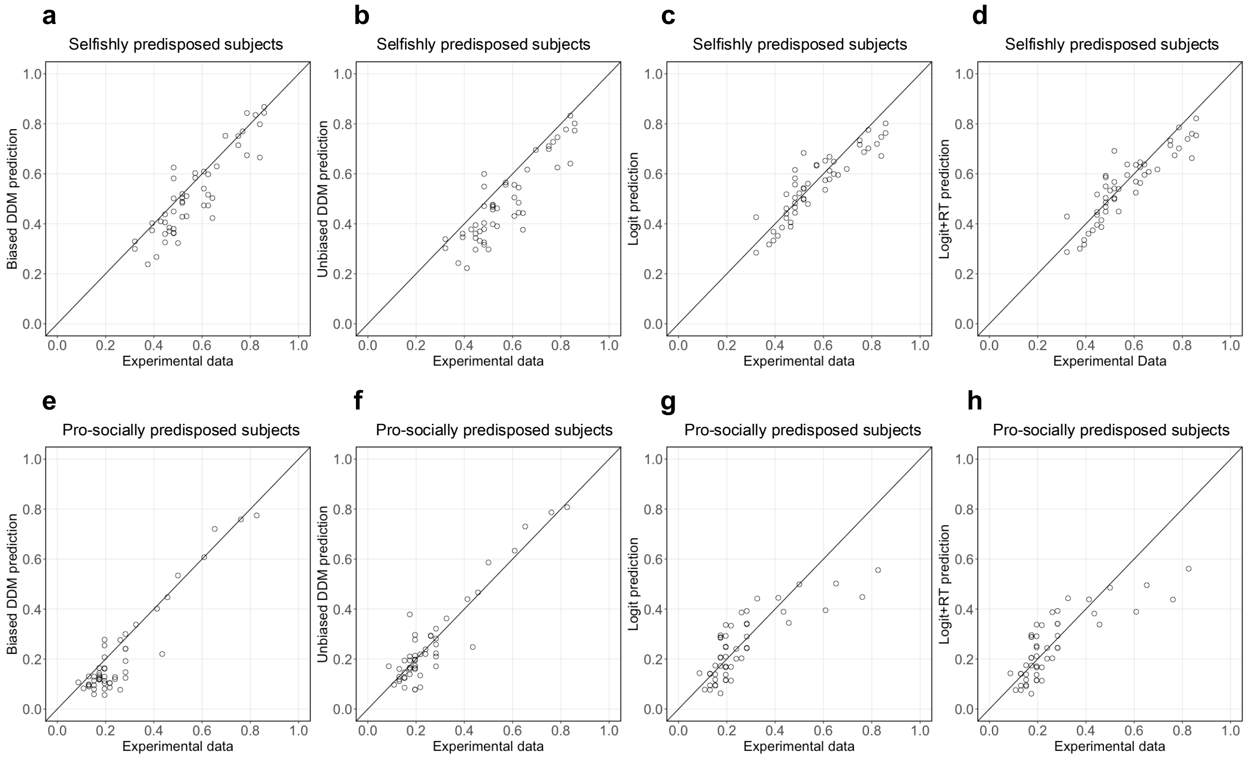

Supplementary Figure 8. Comparisons of the predictions by different models with the experimental data. Here we use Games 51-100 to predict decisions in Games 1-50. Each dot represents one game. X-axis is the probability of selfish choice in the experimental data, and Y-axis is the predicted probability of selfish choice by the biased DDM, the unbiased DDM, the Logit, and the Logit+RT. (**a**) The predicted probability by the biased DDM for selfishly predisposed subjects. (**b**) The predicted probability by the unbiased DDM for selfishly predisposed subjects. (**c**) The predicted probability by the Logit for selfishly predisposed subjects. (**d**) The predicted probability by the Logit+RT for selfishly predisposed subjects. (**e**) The predicted probability by the biased DDM for pro-socially predisposed subjects. (**f**) The predicted probability by the unbiased DDM for pro-socially predisposed subjects. (**g**) The predicted probability by the Logit for pro-socially predisposed subjects. (**h**) The predicted probability by the Logit+RT for pro-socially predisposed subjects.

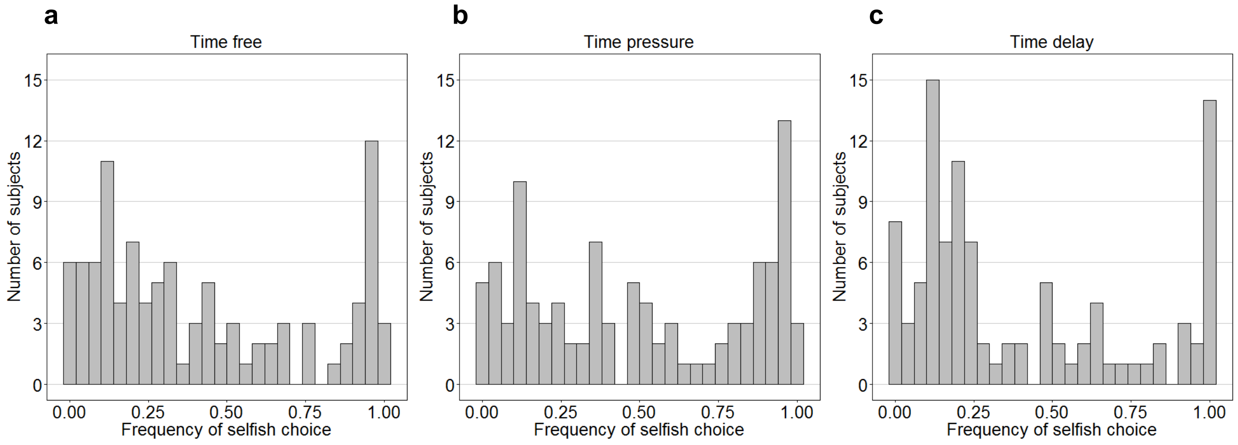

Supplementary Figure 9. The distributions of the frequency of selfish decisions in the three time conditions. (**a**) the time-free condition, (**b**) the time-pressure condition, and (**c**) the time-delay condition.

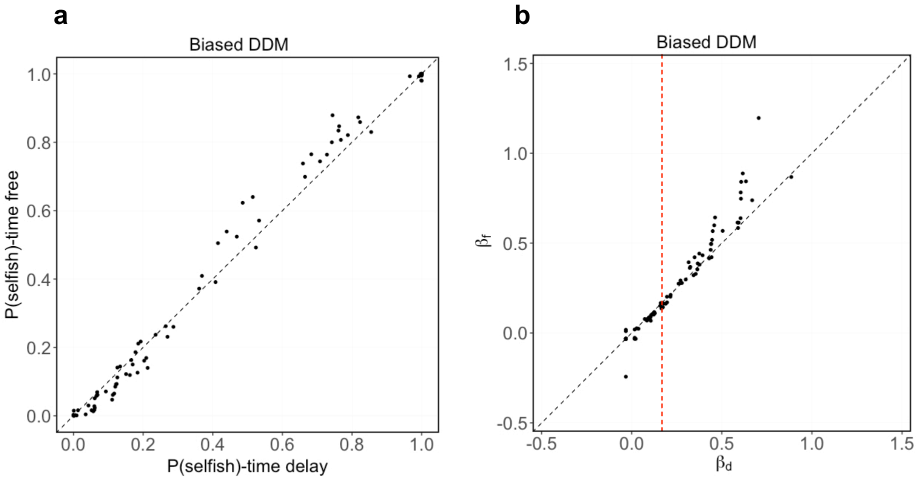

Supplementary Figure 10. The biased DDM simulations in the time-free and time-delay conditions. In the simulations, we use the parameters estimated from the experimental data. (**a**) the simulated probability of choosing the selfish option in the time-free and time-delay conditions, (**b**) the advantageous inequality aversion (β) estimated from the simulated data in the biased DDM simulations (data for plot **a**). We carried out the simulations using the parameters of the 86 subjects whose $\beta_{\mathrm{f}} \in [-0.5, 1]$. We used β_{f} to calculate the utility difference (UD) between choice options, and let the drift rate (dr) be proportional to UD, that is, $dr = 0.2 * UD$. We used the threshold (a) estimated from the experimental data in the time-free simulations, and we used $2a$ in the time-delay simulations. Each dot represents one subject. The black dotted line is the identity line. Note that in (**a**) subjects' choices become more extreme under time free in the biased DDM simulations (two-sided Wilcoxon signed rank tests, $P < 0.001$ for both selfish and pro-social subjects (split by the median indifference β)) compared to time-delay conditions, in (**b**) subjects to the left of the vertical red line (split by the median indifference β, selfish subjects) are consistently shifted downwards while those to the right (pro-social subjects) are consistently shifted upwards in the biased DDM simulations (two-sided Wilcoxon signed rank tests, $P < 0.001$ for both selfish and pro-social subjects). Therefore, the pattern produced by the biased DDM simulations in the time-free and time-delay conditions (**b**) is consistent with the results observed in the experimental data (Fig. 2**b**).

Supplementary Tables

Supplementary Table 1. Logit regression on time condition

Intercept	0.405
	(0.323)
Pro-social	-0.665
	(0.412)
Pressure	-1.504***
	(0.487)
Pro-social × Pressure	2.223***
	(0.609)
AIC	274.504
BIC	287.777
Log Likelihood	-133.252

Notes: The dependent variable is a dummy which indicates whether the subject became more pro-social ($1: \beta_p > \beta_f$ or $\beta_d > \beta_f$) or selfish ($0: \beta_p < \beta_f$ or $\beta_d < \beta_f$) compared to the time-free condition. The independent variable Pro-social is a dummy which indicates whether the subject is prosocial or selfish (split according to the median indifference β). Pressure is a dummy which indicates whether the time condition is time pressure or time delay.
****P* < 0.005, ***P* < 0.01, **P* < 0.05.

Supplementary Table 2. Regressions of $(\beta_p - \beta_d)$ on (β_f −median(indifference β))

	OLS	Logit
Intercept	-0.077	-0.681**
	(0.043)	(0.260)
β_f −median (indifference β)	0.518***	3.048***
	(0.159)	(0.896)
Num. obs	69	90

Notes: In the OLS regression, the dependent variable is $\beta_p - \beta_d$. In the Logit regression, the dependent variable is a dummy which indicates whether $\beta_p > \beta_d$ (1) or $\beta_p < \beta_d$ (0). 33 subjects whose β_f, β_p, or β_d are outside of [-1, 2] are not included in the OLS regression. 12 subjects whose β_f are outside of [-1, 2] are not included in the Logit regression.
****P* < 0.005, ***P* < 0.01, **P* < 0.05.

Supplementary Table 3. Regressions of β change on β_f

	$(\beta_p - \beta_d)$		$(\beta_p - \beta_f)$		$(\beta_f - \beta_d)$	
	OLS	Logit	OLS	Logit	OLS	Logit
	(1)	(2)	(3)	(4)	(5)	(6)
Intercept	-0.163***	-1.190***	-0.077	-0.923**	-0.087***	-0.480
	(0.055)	(0.353)	(0.061)	(0.332)	(0.018)	(0.308)
β_f	0.518***	3.048***	0.092	2.496**	0.425***	1.804*
	(0.159)	(0.896)	(0.175)	(0.835)	(0.053)	(0.773)
Num. obs.	69	90	69	90	69	90

Notes: In OLS regressions, the dependent variable is $\beta_p - \beta_f$, $\beta_f - \beta_d$, or $\beta_p - \beta_d$. In Logit regressions, the dependent variable is a dummy which indicates whether $\beta_p > \beta_f$ (1) or $\beta_p < \beta_f$ (0) under time pressure, whether $\beta_f > \beta_d$ (1) or $\beta_f < \beta_d$ (0) under time delay, and whether $\beta_p > \beta_d$ (1) or $\beta_p < \beta_d$ (0) across time-pressure and time-delay conditions. 33 subjects whose β_p, β_f, and β_d are outside of [-1, 2] are not included in the OLS regressions. 12 subjects whose β_f are outside of [-1, 2] are not included in the Logit regressions.
****P* < 0.005, ***P* < 0.01, **P* < 0.05.

Supplementary Table 4. Estimation results of the biased DDM at the individual level

Parameter	Subject type	Min	1st Qu.	Median	Mean	se	3rd Qu.	Max
Relative starting point (z)	Selfishly predisposed	0.356	0.490	0.572	0.564	0.015	0.631	0.791
	Pro-socially predisposed	0.180	0.352	0.432	0.451	0.019	0.540	0.837
Threshold (a)	Selfishly predisposed	2.013	2.869	3.157	3.356	0.107	3.964	5.462
	Pro-socially predisposed	2.013	3.220	4.145	4.204	0.216	4.858	9.756
Non-decision time (t_0) [s]	Selfishly predisposed	0.243	0.507	0.637	0.743	0.056	0.797	2.882
	Pro-socially predisposed	0.220	0.484	0.644	0.692	0.042	0.863	1.554
Constant (d_c) [s^{-1}]	Selfishly predisposed	-1.132	-0.210	0.066	0.149	0.092	0.430	2.392
	Pro-socially predisposed	-0.890	-0.381	-0.123	-0.089	0.068	0.142	1.239
Weight on own payoff (d_d) [s^{-1}]	Selfishly predisposed	-0.037	0.018	0.045	0.055	0.008	0.080	0.236
	Pro-socially predisposed	-0.043	-0.006	0.027	0.034	0.008	0.055	0.178
Weight on other's payoff (d_r) [s^{-1}]	Selfishly predisposed	-0.005	0.004	0.011	0.013	0.001	0.022	0.035
	Pro-socially predisposed	-0.010	0.012	0.016	0.017	0.002	0.024	0.044

Notes: The starting points of pro-socially predisposed subjects are significantly less than those of selfishly predisposed subjects (P < 0.001, one-sided Mann–Whitney U test). The thresholds of pro-socially predisposed subjects are significantly higher than those of selfishly predisposed subjects (P < 0.001). The non-decision times of pro-socially predisposed subjects are not significantly different from those of selfishly predisposed subjects (P = 0.933, two-sided Mann-Whitney U test). The parameter d_c of selfishly predisposed subjects is significantly less than that of pro-socially predisposed subjects (P = 0.027, one-sided Mann-Whitney U test). The parameter d_d of pro-socially predisposed subjects is significantly less than that of selfishly predisposed subjects (P = 0.031). The parameter d_r of pro-socially predisposed subjects is significantly greater than that of selfishly predisposed subjects (P = 0.024).

Supplementary Table 5. Logit regressions on the biased DDM parameters

	Sign($\beta_p - \beta_d$)	Sign($\beta_p - \beta_f$)	Sign($\beta_f - \beta_d$)
Starting Point (z)	-6.502**	-6.724***	1.464
	(2.367)	(2.349)	(1.926)
Threshold (a)	0.574	0.146	-0.086
	(0.307)	(0.290)	(0.211)
Non-Decision Time (t_0)	-1.356	-1.281	0.125
	(0.795)	(0.776)	(0.635)
Weight on Other's Payoff (d_d)	-3.365	-34.060	-25.784
	(4.434)	(25.694)	(22.866)
Weight on Own Payoff (d_r)	-13.136	-0.872	1.787
	(26.344)	(4.243)	(4.073)
Constant of the Drift Rate (d_c)	-0.416	-0.241	-0.517
	(0.447)	(0.408)	(0.395)
Intercept	1.984	3.247	-0.967
	(1.862)	(1.919)	(1.613)
Num. obs.	102	102	102

Notes: The dependent variable is a binary variable which indicates the sign of the behavioral change across time-pressure and time-delay conditions (1: $\beta_p - \beta_d > 0$; 0: $\beta_p - \beta_d < 0$), the sign of the behavioral change across time-pressure and time-free conditions (1: $\beta_p - \beta_f > 0$; 0: $\beta_p - \beta_f < 0$), and the sign of the behavioral change across time-free and time-delay conditions (1: $\beta_f - \beta_d > 0$; 0: $\beta_f - \beta_d < 0$).
***$P < 0.005$, **$P < 0.01$, *$P < 0.05$.

Supplementary Table 6. Out-of-sample prediction results

	Selfishly predisposed subjects	Pro-socially predisposed subjects
$\sum AE_{\text{biased DDM}}$	3.338	3.115
$\sum AE_{\text{unbiased DDM}}$	4.524	2.284
$\sum AE_{\text{Logit}}$	2.684	3.590
$\sum AE_{\text{Logit+RT}}$	2.688	3.656
Cramer's λ of biased DDM	0.195	
Cramer's λ of unbiased DDM	0.166	
Cramer's λ of Logit	0.164	
Cramer's λ of Logit+RT	0.163	

Notes: In this table, we use the data from Games 51-100 to predict decisions in Games 1-50 in the time-free condition.

Supplementary Table 7. Estimation results of the unbiased DDM at the individual level

Parameter	Subject type	Min	1st Qu.	Median	Mean	se	3rd Qu.	Max
Threshold (a)	Selfishly predisposed	2.019	2.777	3.083	3.298	0.106	3.966	5.436
	Pro-socially predisposed	2.000	3.087	4.025	3.842	0.138	4.598	5.501
Non-decision time (t_0) [s]	Selfishly predisposed	0.206	0.433	0.543	0.661	0.057	0.752	2.763
	Pro-socially predisposed	0.093	0.383	0.551	0.581	0.044	0.733	1.482
Constant (d_c) [s^{-1}]	Selfishly predisposed	-1.126	-0.161	0.112	0.248	0.093	0.600	2.374
	Pro-socially predisposed	-0.866	-0.488	-0.158	-0.133	0.075	0.046	1.396
Weight on own payoff (d_d) [s^{-1}]	Selfishly predisposed	-0.042	0.012	0.045	0.054	0.008	0.080	0.236
	Pro-socially predisposed	-0.052	-0.006	0.026	0.033	0.008	0.057	0.173
Weight on other's payoff (d_r) [s^{-1}]	Selfishly predisposed	-0.006	0.005	0.012	0.013	0.001	0.022	0.035
	Pro-socially predisposed	-0.010	0.010	0.016	0.017	0.002	0.024	0.044

Notes: The thresholds of pro-socially predisposed subjects are significantly higher than those of selfishly predisposed subjects (P = 0.002, one-sided Mann–Whitney U test). The non-decision times of pro-socially predisposed subjects are not significantly different from those of selfishly predisposed subjects (P = 0.635, two-sided Mann-Whitney U test). The parameter d_c of pro-socially predisposed subjects is significantly less than that of selfishly predisposed subjects (P = 0.001, one-sided Mann-Whitney U test). The parameter d_d of pro-socially predisposed subjects is significantly less than that of selfishly predisposed subjects (P = 0.030). The parameter d_r of pro-socially predisposed subjects is significantly greater than that of selfishly predisposed subjects (P = 0.022).

Supplementary Table 8. Estimation results for the biased DDM and the unbiased DDM at the group level

		Subject type	Relative starting point (z)	Non-decision time (t_0) [s]	Threshold (a)	Inter-trial variability of z (szr)
biased DDM	Games 1-50	Selfishly predisposed	0.525 (0.014)	0.455 (0.071)	3.292 (0.055)	0.557 (0.079)
		Pro-socially predisposed	0.411 (0.013)	0.352 (0.039)	3.902 (0.059)	0.208 (0.067)
	Games 51-100	Selfishly predisposed	0.541 (0.015)	0.225 (0.078)	3.432 (0.058)	0.478 (0.091)
		Pro-socially predisposed	0.437 (0.007)	0.581 (0.033)	3.576 (0.044)	0.318 (0.025)
unbiased DDM	Games 1-50	Selfishly predisposed	N/A	0.542 (0.042)	3.269 (0.057)	0.625 (0.038)
		Pro-socially predisposed	N/A	0.135 (0.037)	4.126 (0.085)	0.483 (0.057)
	Games 51-100	Selfishly predisposed	N/A	0.296 (0.078)	3.260 (0.058)	0.387 (0.091)
		Pro-socially predisposed	N/A	0.521 (0.033)	3.468 (0.044)	0.314 (0.025)

Notes: In the unbiased DDM estimation, we fixed the relative starting point at 0.5. The standard errors of the estimators are calculated using a jackknife method[5, 6] and reported in parentheses. N/A, not applicable.

Supplementary Table 9. Logit regressions on half of the data in time-free condition

	Games 1-50				Games 51-100			
Predisposition Type	Selfish	Pro-social	Selfish	Pro-social	Selfish	Pro-social	Selfish	Pro-social
(Intercept)	0.415***	-0.679***	1.011***	-0.695***	0.338***	-0.744***	1.172***	-0.614***
	(0.114)	(0.141)	(0.131)	(0.150)	(0.113)	(0.144)	(0.136)	(0.159)
DicDiff	0.114***	0.096***	0.119***	0.096***	0.114***	0.108***	0.123***	0.110***
	(0.014)	(0.018)	(0.014)	(0.018)	(0.014)	(0.018)	(0.015)	(0.018)
ReceDiff	0.027***	0.035***	0.030***	0.035***	0.026***	0.038***	0.032***	0.039***
	(0.002)	(0.003)	(0.002)	(0.003)	(0.002)	(0.003)	(0.002)	(0.003)
RT			-0.189***	0.004			-0.264***	-0.035
			(0.019)	(0.012)			(0.022)	(0.019)
AIC	3635.547	2465.935	3533.524	2467.840	3659.041	2380.156	3487.787	2378.495
BIC	3653.360	2483.157	3557.274	2490.803	3676.853	2397.378	3511.537	2401.458
Num. obs.	2800	2300	2800	2300	2800	2300	2800	2300

Notes: The dependent variable is a dummy variable which indicates whether the choice is selfish (1) or pro-social (0). The independent variables are the difference between the dictator's payoffs (*DicDiff*), the difference between the receiver's payoffs (*ReceDiff*), and the response time (RT). The models are estimated for selfishly predisposed and pro-socially predisposed subjects separately. The robust standard errors are clustered on each subject and reported in parentheses.
***P < 0.005, **P < 0.01, *P < 0.05.

Supplementary References

1. Fischbacher U. z-Tree: Zurich toolbox for ready-made economic experiments. *Exp. Econ.* **10**, 171-178 (2007).

2. Greiner B. Subject pool recruitment procedures: organizing experiments with ORSEE. *J. Econ. Sci. Assoc.* **1**, 114-125 (2015).

3. Charness G., Rabin M. Understanding social preferences with simple tests. *Q. J. of Econ.* **117**, 817-869 (2002).

4. Fehr E., Schmidt K. M. A theory of fairness, competition, and cooperation. *Q. J. of Econ.* **114**, 817-868 (1999).

5. Quenouille M. H. Approximate tests of correlation in time-series. *J. Royal Stat. Soc. Ser. B Stat. Methodol.* **11**, 68-84 (1949).

6. Tukey J. W. Bias and confidence in not-quite large samples. *Ann. Math. Stat.* **29**, 614 (1958).
